# Multi-site evaluation of the LN34 pan-lyssavirus real-time RT-PCR assay for post-mortem rabies diagnostics

**DOI:** 10.1371/journal.pone.0197074

**Published:** 2018-05-16

**Authors:** Crystal M. Gigante, Lisa Dettinger, James W. Powell, Melanie Seiders, Rene Edgar Condori Condori, Richard Griesser, Kenneth Okogi, Maria Carlos, Kendra Pesko, Mike Breckenridge, Edson Michael M. Simon, Maria Yna Joyce V. Chu, April D. Davis, Scott J. Brunt, Lillian Orciari, Pamela Yager, William C. Carson, Claire Hartloge, Jeremiah T. Saliki, Susan Sanchez, Mojgan Deldari, Kristina Hsieh, Ashutosh Wadhwa, Kimberly Wilkins, Veronica Yung Peredo, Patricia Rabideau, Nina Gruhn, Rolain Cadet, Shrikrishna Isloor, Sujith S. Nath, Tomy Joseph, Jinxin Gao, Ryan Wallace, Mary Reynolds, Victoria A. Olson, Yu Li

**Affiliations:** 1 Poxvirus and Rabies Branch, Division of High Consequence Pathogens and Pathology, National Center for Emerging and Zoonotic Infectious Diseases, Centers for Disease Control and Prevention, Atlanta, Georgia, United States of America; 2 Bureau of Laboratories, Pennsylvania Department of Health, Exton, Pennsylvania, United States of America; 3 Rabies Unit, Wisconsin State Laboratory of Hygiene, Madison, Wisconsin, United States of America; 4 Rabies Laboratory, Center for Zoonotic and Vectorborne Diseases, Maryland Department of Health, Baltimore, Maryland, United States of America; 5 Scientific Laboratory Division, New Mexico Department of Health, Santa Fe, New Mexico, United States of America; 6 Special Pathogens Laboratory, Department of Health, Research Institute for Tropical Medicine, Alabang Muntinlupa City, Manila, Philippines; 7 Rabies Laboratory, Wadsworth Center, New York State Department of Health, Albany, New York, United States of America; 8 Athens Veterinary Diagnostic Laboratory, University of Georgia, Athens, Georgia, United States of America; 9 California Department of Public Health, Sacramento, California, United States of America; 10 Rabies section, Viral Disease, Public Health Institute of Chile, Santiago, Chile; 11 Public Health Command Europe, Laboratory Sciences, Biological Analysis Division, Kirchberg Kaserne, Landstuhl, Germany; 12 Ministère de l’Agriculture, Port-au-Prince, Haiti; 13 OIE Twinned KVAFSU-CVA-Crucell Rabies Diagnostic Laboratory, Deptartment of Veterinary Microbiology, Veterinary College, KVAFSU, Hebbal, Bangalore, India; 14 Animal Health Centre, Ministry of Agriculture, Abbotsford, British Columbia, Canada; University of Helsinki, FINLAND

## Abstract

Rabies is a fatal zoonotic disease that requires fast, accurate diagnosis to prevent disease in an exposed individual. The current gold standard for post-mortem diagnosis of human and animal rabies is the direct fluorescent antibody (DFA) test. While the DFA test has proven sensitive and reliable, it requires high quality antibody conjugates, a skilled technician, a fluorescence microscope and diagnostic specimen of sufficient quality. The LN34 pan-lyssavirus real-time RT-PCR assay represents a strong candidate for rabies post-mortem diagnostics due to its ability to detect RNA across the diverse *Lyssavirus* genus, its high sensitivity, its potential for use with deteriorated tissues, and its simple, easy to implement design. Here, we present data from a multi-site evaluation of the LN34 assay in 14 laboratories. A total of 2,978 samples (1,049 DFA positive) from Africa, the Americas, Asia, Europe, and the Middle East were tested. The LN34 assay exhibited low variability in repeatability and reproducibility studies and was capable of detecting viral RNA in fresh, frozen, archived, deteriorated and formalin-fixed brain tissue. The LN34 assay displayed high diagnostic specificity (99.68%) and sensitivity (99.90%) when compared to the DFA test, and no DFA positive samples were negative by the LN34 assay. The LN34 assay produced definitive findings for 80 samples that were inconclusive or untestable by DFA; 29 were positive. Five samples were inconclusive by the LN34 assay, and only one sample was inconclusive by both tests. Furthermore, use of the LN34 assay led to the identification of one false negative and 11 false positive DFA results. Together, these results demonstrate the reliability and robustness of the LN34 assay and support a role for the LN34 assay in improving rabies diagnostics and surveillance.

## Introduction

Rabies is a fatal zoonotic disease caused by the negative strand RNA virus, *Rabies lyssavirus* (RABV), and similar viruses from the *Lyssavirus* genus (family *Rhabdoviridae*). Rabies is preventable in humans and animals by vaccination. If exposure occurs in humans, prompt administration of post-exposure prophylaxis can reliably prevent rabies, but there is no proven cure once symptoms have developed [1–3]. Given a case fatality rate approaching 100%, timely and reliable rabies diagnosis in a suspected animal is critical to prevent the disease in an exposed individual. The gold standard for post-mortem rabies diagnostics is the direct fluorescence antibody test (DFA or FAT). The DFA test has proven reliable and sensitive for detection of lyssavirus infection and is currently approved for rabies diagnostics of post-mortem brain samples by the World Health Organization (WHO) and World Organization for Animal Health (OIE) [2,4]. As with any diagnostic assay, the DFA test does have several notable limitations. The accuracy of the DFA test relies on the availability of high quality antibody conjugates and the skill of the technicians performing the test, principally their ability to distinguish positive rabies antigen from nonspecific fluorescence. In some cases, DFA test results are inconclusive for a given sample due to high levels of non-specific fluorescence or sample condition. The DFA test requires strict maintenance of fresh tissue at cold chain temperatures, which make the DFA test impractical for rabies diagnostics in field surveillance, remote areas or areas with limited resources.

For years, the DFA test has been supplemented by reverse transcription PCR (RT-PCR) as a secondary, confirmatory test for rabies. However, to date no PCR-based assay has been adopted by the international rabies community for primary diagnosis of animal rabies based on post-mortem brain samples due to concerns about diagnostic accuracy, the ability of labs to perform molecular assays, the detection of all viruses that cause rabies, and/or incomplete validation of the assays currently available [2,4]. Real-time PCR-based diagnostic methods have the potential to improve rabies diagnostics because of their high sensitivity, fast turn-around time, and objective diagnostic thresholds. Real-time PCR-based tests require a real-time PCR machine as well as training and experience performing molecular diagnostic tests. However, many diagnostic laboratories worldwide are using real-time PCR assays for routine diagnosis of a vast variety of infectious diseases, so the cost and training required to implement a new rabies-specific assay is minimal. One potential advantage over antigenic-based tests is that samples can be stored at room temperature in nucleic acid stabilization buffers [5–7], which may allow for testing of samples that cannot be maintained frozen until testing. Reports of new, low cost portable PCR diagnostic systems [8,9] also highlight the potential of PCR assays in the field or remote areas.

Rabies RT-PCR assays have undergone rapid evolution over the last two decades [10,11]. Traditional RT-PCR assays have proven reliable at detecting rabies virus RNA but can be prone to false positive results and require additional sequencing steps to confirm positive results [10,11]. These additional steps can be lengthy and impractical in resource-limited areas or regions with high rabies burden. Several real-time (also called quantitative) RT-PCR rabies diagnostic assays have been developed that exhibit low false positive rates while maintaining high sensitivity [10–20]. In general, Taqman probe-based assays have increased specificity comparted to SYBR green assays, but the significant sequence diversity among the *Lyssavirus* genus [21] has made it difficult to develop a single, robust, easy-to-use Taqman-based assay for rabies diagnostics. Several rabies real-time RT-PCR diagnostic assays have been validated to detect a subset of lyssaviruses [14,15,22,23], yet few published assays cover the breadth of known lyssaviruses [16,24,25]. The Taqman-based LN34 assay [16] is a strong candidate for rabies diagnostics due to its high sensitivity, its ability to detect across the diverse *Lyssavirus* genus in a single assay set-up, and its simple, easy-to-implement design.

The LN34 assay amplifies and detects a region at the beginning of the genome that is conserved across divergent lyssavirus species, the leader sequence and part of the nucleoprotein (N) gene [16]. A combination of degenerate primers and a multiplexed, locked nucleic acid Taqman probe increase the broad reactivity of the assay. The primer and probe binding sites are highly conserved across lyssaviruses, and the LN34 assay was validated using a panel of 88 diverse lyssavirus isolates [16]. The LN34 assay includes an artificial RNA that acts as an analyte-positive control, allowing for standardization and optimization across laboratories and between runs to ensure assay sensitivity and robustness. The LN34 assay also includes a host-species control β-actin real-time RT-PCR assay adapted from Wakeley et al. (2005) [15] that targets host β-actin mRNA, spanning exons 4 and 5, which is conserved across mammal species. The β-actin assay ensures the presence of RNA in each sample and acts as an indicator of PCR inhibition, failed extraction or RNA degradation.

Here, we present the results of the largest evaluation of a rabies real-time RT-PCR diagnostic assay. A total of 2,978 post-mortem brain samples from rabies suspect animals originating from Africa, Asia, the Americas, Europe, and the Middle East were tested, including 1,049 DFA positive samples. Fourteen laboratories from seven countries participated. LN34 assay results were compared to results of the gold standard DFA test to estimate diagnostic sensitivity and specificity. Features of the LN34 assay such as variability, robustness, sensitivity, specificity, and ease of implementation in new laboratories were investigated. A detailed protocol and diagnostic guidelines are provided for implementation of the LN34 assay in any laboratory with real-time RT-PCR capabilities and experience.

## Materials and methods

### Samples

Post-mortem brain tissue samples were obtained via routine surveillance or diagnostic service activity of the Centers for Disease Control and Prevention Poxvirus and Rabies Branch (CDC; Atlanta, GA, USA); Pennsylvania Department of Health (Exton, PA, USA); Wisconsin State Laboratory of Hygiene (Madison, WI, USA); Maryland Department of Health (Baltimore, MD, USA); New Mexico Department of Health (Santa Fe, NM, USA); Research Institute for Tropical Medicine (Alabang Muntinlupa City, Manila, Philippines); New York State Department of Health (Albany, NY, USA); University of Georgia (Athens, GA, USA); California Department of Public Health (Sacramento, CA, USA); Public Health Institute of Chile (Santiago, Chile); Public Health Command Europe (Kirchberg Kaserne, Landstuhl, Germany); Ministry of Agriculture (Port-au-Prince, Haiti); Karnataka Veterinary, Animal and Fisheries Sciences University (Hebbal, Bangalore, India); or Ministry of Agriculture (Abbotsford, BC, Canada). No animal or human sampling was performed for this study. All diagnoses and clinical outcomes were based on DFA results or as per the normal operation of the participating laboratory.

### RNA extraction and real-time RT-PCR

In most cases, RNA extraction were performed in accordance with the protocol available at https://www.protocols.io/private/5c970341ebdf05cba17e58ebc16dff08. Other RNA extraction methods and kits used successfully in the pilot study include traditional phenol-chloroform extraction, PureLink RNA Mini Kit (Invitrogen), RNeasy FFPE kit (Qiagen), and the QIAamp RNA Mini kit (Qiagen). Three laboratories were successful in implementing automated extraction using the QIAsymphony (Qiagen), NucliSens easyMAG (bioMérieux) and EZ1 RNA Mini kit (Qiagen). Each of these kits was validated against the provided RNA extraction kit prior to use with the LN34 assay.

Real-time RT-PCR was performed as described previously [16], and a detailed, updated protocol is available at https://www.protocols.io/private/86d245bf034439795301b79dda52ee96. Briefly, each sample was tested by two single-step RT-PCR assays (LN34 and β-actin): one that amplifies and detects the lyssavirus RNA genome and one that amplifies and detects host β-actin mRNA. PCR guidelines suggest each sample be run in triplicate and that each assay run include both analyte positive (artificial RNA) and negative (no template) control reactions in triplicate for each assay run.

LN34 and β-Actin assay primers and probes were generated at CDC (Table 1). Primers were diluted to 10 μM, and probes were diluted to 5 μM in TE buffer (10 mM Tris (pH 8.0), 10 mM EDTA). The artificial positive control RNA was produced by in vitro transcription, followed by DNase treatment and purification per the manufacturer’s instructions (MEGAshortscript^™^ T7 Transcription Kit, Invitrogen). Positive control RNA was diluted in RNA stability buffer (THE Ambion RNA Storage Solution, Invitrogen) to produce a Ct value of approximately 25 in the LN34 assay.

**Table 1:** Primers and probes used in the LN34 real-time RT-PCR assay.

Name	Sequence
LN34 Forward Primer 1	ACGCTTAACAACCAGATCAAAGAA
LN34 Forward Primer 2	ACGCTTAACAACAAAATCADAGAAG
LN34 Reverse Primer	CMGGGTAYTTRTAYTCATAYTGRTC
LN34 Probe	(FAM) AA+C+ACCY+C+T+ACA+A+TGGA (BHQ1)
β-Actin Forward Primer	CGATGAAGATCAAGATCATTGC
β-Actin Reverse Primer	AAGCATTTGCGGTGGAC
β-Actin Probe	(HEX)-TCC ACC TTC CAG CAG ATG TGG ATC A-(BHQ1)
Positive Control RNA	GCA CAG GGT ACT TGT ACT CAT ACT GAT CTG AAT CCA TTG TAG AGG TGT TAG AGC ACG ACA GGT TTC CCG ACT GGA TCT TTC TTT GAT CTG GTT GTT AAG CGT TCG CCC TAT AGT GAG TCG TAT TAC A

LN34 probe is labeled by fluorescent FAM at the 5ʹend and Black Hole quencher (BHQ1) at the 3ʹend. Locked nucleotide modified bases are indicated by a plus preceding the base in the sequence (e.g. +A, +G, +C, +T). β-actin probe is labeled by fluorescent HEX at the 5ʹend and Black Hole quencher (BHQ1) at the 3ʹend.

### Direct fluorescent antibody (DFA) test

All samples were tested for the presence of rabies virus antigen following standard protocol for the direct fluorescence antibody (DFA) test in the testing laboratory (for example, [4,26–28]). Guidance on the DFA procedure was not part of the LN34 international assay evaluation, and each laboratory was encouraged to use their current approved, working DFA/FAT protocol for rabies diagnostics. Thus, specific details about the DFA procedure for each laboratory including antibody conjugates used and conjugate dilution were not controlled across the 14 participating labs. The DFA test and LN34 assay were performed in the same laboratory for each sample. Previously reported DFA results were used for archived samples.

### Limit of detection analysis

The limit of detection of the LN34 assay was estimated using serial dilutions of positive control RNA. Artificial positive control RNA was generated by in vitro transcription as described above. Twenty tenfold serial dilutions of RNA were then prepared in RNA stability buffer (THE Ambion RNA Storage Solution, Invitrogen). RNA concentration was estimated using Qubit RNA Broad Range and High Sensitivity Assays (Invitrogen) for all RNA dilutions falling within the working range of either assay. Qubit data were extrapolated to estimate the concentration of RNA in all prepared serial dilutions; this concentration data was used to estimate the mass of RNA added to each real-time PCR reaction. RNA mass was converted to copy number using the formula (RNA mass (g)/RNA molecular weight (g/mol)) * 6.023 × 10^23^. Molecular weight was determined to be approximately 40,872 g/mol based on the sequence of the positive control ssRNA. All serial dilutions were then tested by the LN34 real-time PCR assay. Percent success of the assay (based on >20 replicates for each RNA concentration) was plotted against estimated copy number and a sigmoidal curve was fitted to the data using a two parameter log-logistic model in the dose response curves (drc) package in R (drm function, LL.3 with d fixed at 100 and a lower limit of 0) [29]. The analytical sensitivity of the LN34 assay was estimated by the effective dose function (ED) in the drc package [29]) as the amount of RNA producing amplification in 95% of trials.

Tenfold serial dilutions of RNA extracted from lyssavirus positive samples were prepared and tested by the LN34 assay. Six isolates were tested: RABV_1 (south central skunk variant isolated from a horse in USA), RABV_2 (cosmopolitan dog variant (India) isolated from a human in USA), RABV_3 (*Tadarida brasiliensis* variant (southeastern USA) isolated from a human in USA), RABV_4 (strain CVS11), DUVV (isolated from *Miniopterus spp*. in South Africa), and LBV (lineage C, UK Ethiopia). DUVV isolate was provided by ANSES and LBV isolate was provided by OIE as part of inter-laboratory testing panels. RABV strain CVS11 was tested in duplicate; all other samples were tested in triplicate. Average Ct values were plotted against log10(sample dilution). Samples that produced no amplification were assigned a Ct value of 45 for graphing purposes. Linear regression analysis was performed for each sample using the linear model (lm) function in the R Stats Package v3.3.1 [30].

### Repeatability and reproducibility studies

Repeatability within an assay run: Within sample variation was tested using 150 (106 DFA positive) samples tested as part of confirmatory testing at the CDC during 2017Samples were chosen to cover the entire LN34 assay Ct value range, especially at Ct > 30. LN34 and β-actin assays were performed for each sample in triplicate. Ct value of replicate 1 was plotted against replicate 2; a line was then fitted to the resultant dataset by linear regression using the linear model (lm) function in the R Stats Package v3.3.1 [30]. To evaluate if variability between replicates was dependent on Ct value, average standard deviation was plotted against average Ct value for each sample.

Repeatability between assay runs: A panel of 7 brain samples (5 positive, 2 negative) in TRIzol was used to examine LN34 and β-actin assay repeatability within a laboratory. LN34 Ct values of the samples used ranged from not detected to 35.2; β-actin Ct values ranged from 17.4 to 29.5. Samples with LN34 Ct > 30 were made by diluting positive clinical samples into negative samples 1:2,000 or 1:5,000. All testing was performed in the rabies laboratory at CDC. Each sample was homogenized in TRIzol, made into identical aliquots and extracted at least three times by three operators. Operators were blinded and performed extractions as described in the RNA extraction protocol (https://www.protocols.io/private/5c970341ebdf05cba17e58ebc16dff08). Extracted RNA was then tested for the presence or absence of lyssavirus RNA following the LN34 real-time PCR protocol. To examine inter-assay variability, the positive extracted RNA samples were each tested in triplicate in at least three independent assay runs by different operators on different days. Qualitative diagnostic results were determined based on the diagnostic Ct value cut-offs of 35 for LN34 and 33 for β-actin (see below). Average standard deviation, coefficient of variation, and change in Ct value were calculated to estimate variability in LN34 and β-actin assay Ct values for a given sample.

Reproducibility: A panel of 20 extracted RNA samples (10 positive and 10 negative) was sent to three laboratories for independent testing to assess assay reproducibility. LN34 Ct values ranged from not detected to 29.0; β-actin Ct values ranged from 15.4 to 26.3. Laboratories participating in the reproducibility study included CDC, Pennsylvania Department of Health, and Wisconsin State Laboratory of Hygiene. Samples were extracted, aliquotted, and shipped from CDC to the other two laboratories. Variability in RNA extraction between laboratories was not evaluated. Samples were assigned non-informative identifiers, and all testing personnel were blinded to sample identity and DFA results. Samples were tested in triplicate in the LN34 and β-actin assays in each laboratory. Average standard deviation, coefficient of variation, and range of Ct value were calculated to estimate variability in LN34 and β-actin assay Ct values for a given sample.

Variability between laboratories: A second assessment of assay reproducibility was performed using LN34 assay results for the positive control RNA distributed by CDC. Each pilot study laboratory was advised to run the positive control RNA in triplicate during each assay run. Twelve laboratories reported average Ct values for the positive control RNA for each assay run. The variability in LN34 Ct values for the positive control RNA within a given laboratory as well as between laboratories was evaluated by coefficient of variation and standard deviation. Variability between Roche Light Cycler and ABI Step One Plus or BioRad CFX96 real-time PCR machines in the same laboratory was assessed at the Research Institute for Tropical Medicine (Philippines) using a panel of 14 samples under the same cycle conditions.

### Pilot study

In total, 1,049 DFA positive, 1,848 DFA negative, and 81 DFA inconclusive brain samples were tested in an international multi-laboratory pilot study to evaluate the pan-lyssavirus LN34 assay. Brain tissue samples obtained from moribund animals, dead animals, or animals involved in human rabies exposure were collected during routine surveillance and diagnostic services of the participating laboratories. Each brain sample was tested for both rabies virus antigen by the DFA test and rabies virus RNA using the LN34 assay at one of 14 participating laboratories (Table 2). Laboratories were chosen based on willingness to participate, lack of molecular-based rabies diagnostics, and/or previous experience with PCR. Laboratories were asked to test an equal number of known positive and negative samples. Given a disease prevalence of 50% for the samples tested (artificially controlled), a minimum of 250 positive and 250 negative samples was set to achieve p < 0.05 for diagnostic sensitivity and specificity >90% and power >80% [31]. Each participating laboratory was asked to test at least 150 negative and 150 positive samples, including archived samples, within a six month period with the goal of covering the diversity of host species, lyssavirus diversity, conditions causing similar clinical presentation as rabies, and normal occurrence of rabies suspect cases for each laboratory. Samples included fresh, frozen, archived, deteriorated, and formalin-fixed brain tissue from various non-human mammals.

**Table 2:** List of the laboratories that participated in the LN34 assay evaluation and the country or region of sample origin.

Laboratory	Laboratory Location	Sample Origin
Maryland Department of Health and Mental Hygiene	United States	United States
Pennsylvania Department of Health	United States	United States
New Mexico Department of Health	United States	United States
California Department of Public Health	United States	United States
Wisconsin State Laboratory of Hygiene	United States	United States
Wadsworth Center, New York State Department of Health	United States	United States
University of Georgia	United States	United States
United States Army Public Health Command Europe	Germany	Middle East, Europe
Animal Health Centre, British Columbia	Canada	Canada
Research Institute for Tropical Medicine	Philippines	Philippines
Ministère de l’Agriculture	Haiti	Haiti
Instituto de Salud Pública de Chile	Chile	Chile
Karnataka Veterinary, Animal and Fisheries Sciences University	India	India
Centers for Disease Control and Prevention	United States	Georgia
		Asia
		Ethiopia

Results of the LN34 assay were used only for assay evaluation and were not used to make human rabies post-exposure vaccination treatment decisions. All results were sent to CDC for compilation and analysis. Any discordant or unusual samples were sent to CDC for re-testing by both DFA and LN34 for U.S. laboratories. Discordant samples or unusual samples from non-U.S. laboratories were re-tested and/or re-extracted at the testing laboratory.

All reagents required for the LN34 assay, including real-time RT-PCR protocol and guidance, suggested RNA extraction protocol, AgPath-ID One-Step RT-PCR kit (Applied Biosystems), Direct-zol RNA MiniPrep kit (Zymo) for RNA extraction using TRIzol Reagent (Invitrogen), positive control RNA, and LN34 and β-actin assay primers and probes, were provided to each participating laboratory by CDC. CDC provided guidance and support through conference calls and email communications. CDC provided the testing laboratory in Haiti with a real-time PCR-machine and hands-on training for the LN34 assay. All other participating laboratories had previous experience with real-time PCR and access to at least one real-time PCR machine capable of detecting FAM and HEX/VIC dyes. Excluding Haiti, no other participating laboratory received hands-on training for LN34 assay implementation.

Participating laboratories were encouraged to include descriptive data for each sample, including host species and sample condition. Pilot study data were analyzed to identify if LN34 or β-actin Ct values were affected by sample condition. Only a subset of samples (1,502 total samples, 335 DFA positive) contained sample condition information. Samples were considered to be of good condition if they were described as good, ok, or not a full cross section of brain stem but no other indication. Samples were considered poor condition if they were described as decomposed, containing bacteria, liquefied, poor, smashed, rotten, bloody, autolytic, dried out, desiccated, tacky, decaying, loss of structure, “shot”, compromised, aged, and/or soupy. Samples were categorized as formalin-fixed if it was mentioned. A sample was considered fresh if it was tested within one week of sample collection; samples tested >1 month to 13 years after collection (including 12 archived samples with unknown collection dates) were included in the frozen/archived category. For analysis, “good” was compared to “poor”, “fresh” was compared to “frozen”, and “fixed” was compared to all others.

### Cut-off determination

Diagnostic thresholds for the LN34 assay were determined using Receiver Operator Characteristic (ROC) analysis using the pROC package in R [32]. LN34 Ct value diagnostic cut-off to identify rabies positive samples was estimated using results from DFA positive and negative pilot study samples. LN34 Ct value of 45 was assigned to all samples that did not exhibit amplification that passed the threshold. A threshold that optimized diagnostic sensitivity and specificity was chosen. LN34 assay accuracy was investigated by area under the ROC curve (AUC) analysis.

### Diagnostic specificity/sensitivity

Diagnostic sensitivity was determined as (LN34pos;DFApos)/(total DFA pos) or (LN34pos;DFApos)/((LN34pos;DFApos)+(LN34neg;DFApos)+(LN34inc;DFApos)), where neg = negative, pos = positive, and inc = inconclusive. Diagnostic specificity was determined as (LN34neg;DFAneg)/(total DFA neg) = (LN34neg;DFAneg)/((LN34neg;DFAneg)+(LN34pos;DFAneg) +(LN34inc;DFAneg)). 95% confidence intervals were based on Clopper-Pearson confidence intervals [33]. Only verified results (one result per sample, see sample quality control, below) were used for diagnostic cut-off value determination and diagnostic sensitivity and specificity calculations. All raw results are discussed in detail below.

### Sample quality control

Certain quality criteria were mandated for data to be included in analyses, and data that did not meet these criteria were removed from the final dataset. Unique identifiers were required for all samples. In one laboratory, duplicate identifiers were used for two samples with reported discordant DFA and LN34 results; the presence of multiple samples with the same identifier suggested a possible mistake especially since the DFA and LN34 tests were performed on different days; re-testing was not possible. These data were removed from the dataset. Samples were also removed from the dataset if both LN34 and β-actin results were not reported.

Samples were re-tested if results from all three replicates did not agree. An entire assay run was discarded if the no template control reactions produced amplification or if more than one sample exhibited amplification in only 1/3 or 2/3 replicates. Any sample producing Ct > 38 in 1/3 replicates and no amplification in 2/3 replicates could be called negative. An entire assay run was invalidated if the positive control RNA did not produce amplification. Results from assay runs or replicate wells that exhibited atypical, non-exponential amplification were also discarded. All invalid assays, samples or replicates were marked for repeat testing.

No amplification in the β-actin assay may indicate extraction failure, poor sample quality, PCR inhibition or RNA degradation. Such samples were flagged for re-testing. Discordant samples and samples with Ct values in the range of inconclusive samples based on diagnostic thresholds (LN34 Ct >35 or β-actin Ct > 33 with no amplification in LN34 assay) were subject to re-testing either in the participating laboratory or at CDC. RNA re-extraction was performed in cases where extraction failure or contamination was suspected. Ten or one hundred fold sample dilution was suggested if PCR inhibition was suspected. Samples that failed to amplify or had β-actin Ct > 33 with no LN34 amplification were considered to have insufficient sample for diagnostic evaluation and were not included in the diagnostic cut-off evaluation or diagnostic specificity/sensitivity estimates. The total number of samples that failed to amplify or produced Ct > 33 in the β-actin assay was very low (51 out of 2,839 non-fixed samples or 1.8%), and re-extraction or re-testing of these samples produced β-actin Ct < 33 for all samples sent to CDC for re-testing (29 samples).

A few discordant samples revealed concordant results upon re-testing at the participating laboratory or CDC (S1 Table). These samples were verified as being concordant and the discordant results were not included in any cut-off or diagnostic sensitivity/specificity analyses; only one result per sample was included in all analyses.

### Data analysis

All statistical analyses were performed and all graphs were made in RStudio 0.99.491 [34] using R version 3.3.1 [30]. Beeswarm plots were made using the R package beeswarm; violin plots were made using ggplot2 [35]. Graphs and figures were generated and formatted using Inkscape 0.91.

Pilot study data, repeatability, and reproducibility data were fit to linear mixed-effects models by maximizing log-likelihood using the linear mixed effects function (lme) in the nlme package [36]. Linear mixed-effects models were used to predict Ct value as a function of replicate for within-assay repeatability study, as a function of laboratory for reproducibility study, or as a function of replicate, extraction, and/or assay run for between assay run repeatability study; sample was included as a random term. Linear mixed-effects models were also used to model Ct value as a function of sample condition for pilot study data, with sample as a random term. Linear mixed models were used to model the effect of real-time PCR machine on LN34 Ct value, with sample as a random term.

Models were compared to a null model by likelihood ratio test to determine if the variable examined had a significant effect on Ct value using the anova function in the R Stats Package v3.3.1 [30]. The least-squares mean (lsmeans) function from the lsmeans package [37] was then used to perform pairwise comparisons between groups; p-values were corrected for multiple comparisons using a Bonferroni correction.

All analyses of Ct value variability were limited to samples that exhibited amplification. A cutoff of p < 0.05 was considered significant.

## Results

### LN34 assay analytical sensitivity

**Fig 1:**
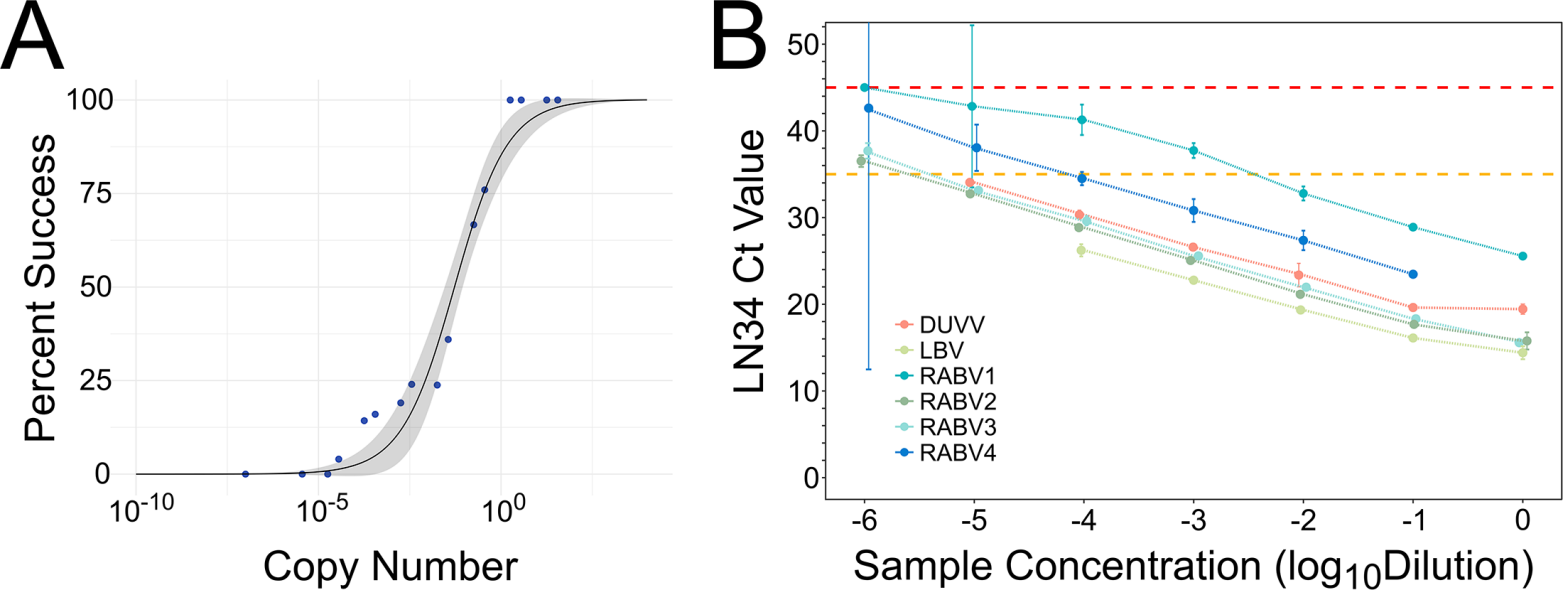
Analytical sensitivity of the LN34 assay. A. Result of limit of detection analysis using serial dilutions of artificial positive control RNA. Estimated RNA copy number was plotted against the percent of runs in which amplification was observed (blue dots). A sigmoidal curve was fit to the data to estimate the limit of detection as the RNA copy number corresponding to 95% assay success (black line, gray shading indicates 95% confidence interval). B. LN34 assay Ct value upon 10 fold serial dilutions of RNA extracted from six lyssavirus isolates. Average Ct value and 95% confidence intervals are shown; several points are artificially offset along the x axis to avoid overlap. Linear regression analysis revealed the following slopes: DUVV -3.58, LBV -3.37, RABV1–3.64, RABV2–3.84, RABV3–3.66, RABV4–3.56. LN34 assay diagnostic cut-off for positive samples (Ct 35) is highlighted in yellow. Assay failure (Ct 45) is shown in red.

A previous report by Wadhwa et al. (2017) demonstrated the high sensitivity of the LN34 real-time RT-PCR assay as well as its ability to detect divergent lyssaviruses [16]. The limit of detection of the LN34 assay was estimated to be on the scale of single digit copies of the genome using droplet digital PCR with rabies virus ERA strain as well as the LN34 assay positive control RNA [16]. Using serial dilutions of the LN34 assay positive control RNA, we confirmed the high sensitivity of the LN34 assay: 95% LN34 assay success corresponded to an estimated 8 RNA copies (95% confidence interval: 0–18 copies, Fig 1A). Furthermore, to determine the sensitivity within true clinical samples, an examination of six viral isolates with LN34 Ct values ranging from 14.6 (very high RNA level) to 25.6 (average RNA level) was conducted. The LN34 assay continued to produce a positive result upon dilution of these RNA samples 100–100,000 times; these samples were still detected upon further dilution, but the result was inconclusive based on a diagnostic cut-off value of Ct 35 for rabies positive samples (see below). The LN34 assay was unable to detect the diluted clinical samples (no amplification) only after 1,000,000 fold dilution (Fig 1B). The average efficiency of the LN34 assay based on these samples was 91.5 ± 2.9% (Fig 1B). We next sought to evaluate potential sources of variability through repeatability and reproducibility studies.

### LN34 assay repeatability

**Fig 2:**
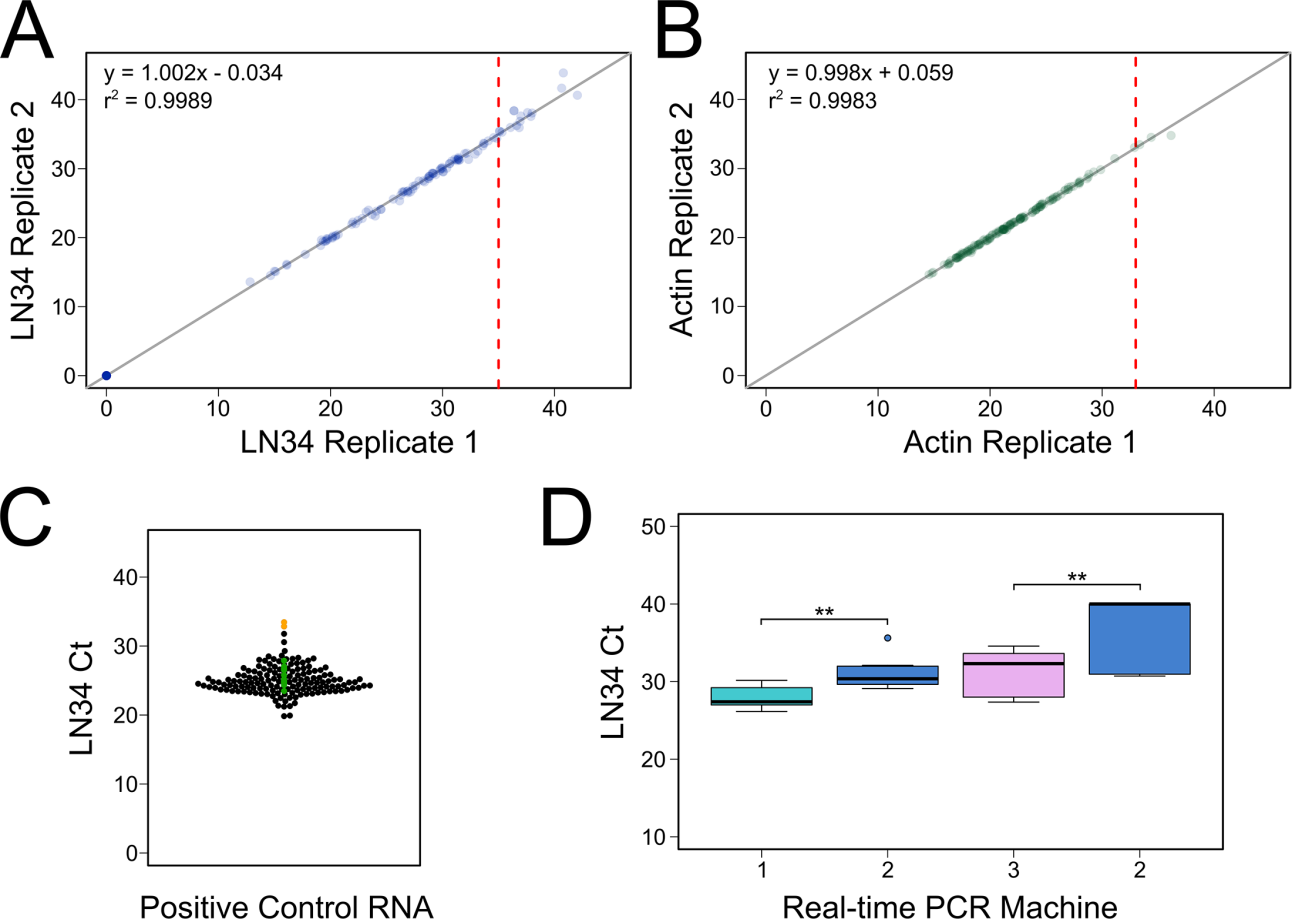
LN34 assay repeatability and reproducibility. A–B. Comparison of replicate Ct values for the same RNA sample tested in the same assay run for LN34 (A) and β-actin (B) assays. Ct value for replicate 1 is plotted against replicate 2. Gray line indicates identity (y = x). Results of linear regression analysis is shown in the upper left corner. Vertical red lines indicate the diagnostic cut-off values for positive samples for each assay. Points are transparent; darker color indicates overlapping points. Samples that failed to amplify are plotted at Ct 0. C. LN34 Ct values reported for positive control RNA tested in 12 laboratories shown as a beeswarm plot. Each dot represents the average value for one assay run; points are plotted according to Ct value (y-axis), then offset along the x-axis to show the distribution of points at each Ct value. Orange dots indicate Ct values observed in one laboratory using a PCR machine with decreased sensitivity, and green dots indicate Ct values reported from the same laboratory using a different PCR machine. D. Comparison of LN34 Ct value for a panel of 14 samples tested in three real-time PCR machines. Machine 2 was determined to produce significantly higher Ct values than either Machine 1 or 3, for the same sample. Boxplots show median (thick line) and 25^th^ and 75^th^ quartiles. Whiskers extend to 1.5×(inter-quartile range); data outside whiskers are plotted individually. ** p < 0.01.

Both the LN34 and β-actin assays displayed a high level of agreement between replicate reactions based on results of 150 samples tested at CDC Atlanta during 2017 (Fig 2A and 2B), and no significant effect of replicate was observed on Ct values for either assay (p = 0.981 LN34, p = 0.975 β-actin, log likelihood test). Furthermore, the variability between replicates was uniform across Ct values corresponding to positive samples (S1 Fig). Larger variability was observed at Ct values close to the assay’s upper limit, Ct > 35 (Figs 2A, 2B, S1A and S1B).

Inter- and intra-assay variability were evaluated by a repeatability study at CDC Atlanta. The qualitative diagnostic results for each sample were 100% in agreement with the original DFA test results. The intra- (between replicates) and inter-assay (between runs and between extractions) variability were determined for each sample using standard deviation, coefficient of variation and average difference in Ct value (Table 3). As expected, slightly more variability was observed between RNA extractions and independent assay runs than between sample replicates (Table 3). However, the difference in Ct values remained small for all conditions. There was no significant effect of replicate or extraction on LN34 Ct value (p = 0.991 and 0.400, respectively, log-likelihood test). A significant effect of assay run was observed (p < 0.001, log-likelihood test), but Ct value differed by only approximately 1 Ct (Table 3). There was also no effect of replicate on β-actin Ct value (p = 0.701), but there was a significant effect of assay run and extraction (p < 0.001, log-likelihood test), with Ct value differing up to 1.5 between assay runs and up to 1.3 between extractions. The between replicate variability observed for these samples was in line with the observed variability in the 150 samples tested at CDC Atlanta. Overall, these repeatability studies revealed low intra- and inter-assay variability, with differences of 0 to 1.5 Ct values.

**Table 3:** Variation between replicate, extraction, assay run, and laboratory observed during repeatability and reproducibility studies.

		Replicate	Extraction	Assay Run	Laboratory
LN34 Assay	CV	0.35 ± 0.06%	0.72 ± 0.27%	1.33 ± 0.32%	9.13 ± 2.06%
	SD	0.09 ± 0.02	0.12 ± 0.07	0.36 ± 0.16	1.89 ± 0.45
	ΔCt	0.18 ± 0.04	0.39 ± 0.12	0.99 ± 0.43	3.70 ± 0.94
β-Actin Assay	CV	0.48 ± 0.13%	2.15 ± 0.75%	1.86 ± 0.70%	6.56 ± 1.00%
	SD	0.10 ± 0.03	0.44 ± 0.12	0.41 ± 0.10	1.34 ± 0.20
	ΔCt	0.18 ± 0.05	0.97 ± 0.28	1.14 ± 0.41	2.57 ± 0.39

Variability shown as average coefficient of variation (CV), standard deviation (SD) and difference in Ct values (ΔCt) with 95% confidence intervals.

### LN34 assay reproducibility

To investigate variability between laboratories, a panel of 20 extracted RNA samples was tested in three different laboratories. LN34 and β-actin Ct values were similar for the same sample across different laboratories, though the observed variability was larger than that observed in the repeatability study (Table 3). Analysis by linear mixed modeling revealed a significant effect of laboratory on LN34 and β-actin Ct value (p < 0.001 for both). These findings are not unexpected as RNA can undergo degradation during shipment and freeze-thaw cycles, and this between laboratory study included variability introduced by different operators, different real-time PCR machines, different reagent lots, and other variables. Despite this effect, the diagnostic results were in complete agreement for all samples tested and all results were concordant with previously reported DFA test results. One laboratory reported PCR failure for two wells in the β-actin assay; both samples exhibited normal amplification upon repeat. The average Ct value for the same sample varied by less than 5 Ct (Table 3).

In addition to the reproducibility study described above, we analyzed the variability in positive control RNA Ct values reported across 12 participating laboratories. Each laboratory received an aliquot of positive control RNA from CDC Atlanta, and the average LN34 Ct value of the positive control RNA was reported for each assay run. Across all laboratories, the average Ct value for the positive control RNA was 25.64 ± 1.22, and the distribution of positive control Ct values showed low variability (Fig 2C). The coefficient of variation was 8% based on 149 data points, similar to the between laboratory coefficient of variability observed for the reproducibility panel (Table 3). Positive control RNA Ct values ranged from 19.84 to 33.45 (13.61 Ct difference); however 95% of the reported Ct values were between Ct 21 and 29 (8 Ct difference). Within a laboratory, the coefficient of variability in positive control RNA Ct value was 5.89 ± 1.15%, similar to the variability observed between assay runs for the repeatability study performed at CDC Atlanta (Table 3).

One laboratory exhibited noticeably higher positive control Ct values during early assay implementation (Fig 2C, orange). This was cause for concern, as high Ct values for positive control RNA could indicate decreased sensitivity of the LN34 assay in this laboratory. Further investigation revealed that the diminished sensitivity could, at least in part, be attributed to a Roche-Light Cycler real-time PCR machine. A side by side comparison of 14 samples performed at the participating laboratory identified a significant decrease in sensitivity (increase in Ct value) using the Roche Light Cycler (Fig 2D, Machine 2) compared to ABI Step One Plus (p = 0026, Machine 1) or the BioRad CFX96 machines (p = 0.0001, Machine 3) in this laboratory. Further testing in this laboratory using either the ABI Step One Plus or the BioRad CFX96 machines produced much lower LN34 Ct values (Fig 2C, green).

These findings establish a baseline of acceptable variability in the LN34 assay and show that use of a standardized positive control RNA is critical during assay implementation to ensure laboratories and real-time PCR machines meet the sensitivity requirements of the LN34 assay.

### Multi-site, large-scale comparison of the LN34 assay and the DFA test

The LN34 assay was evaluated and compared to the gold standard DFA test during a multi-site evaluation study taking place during 2016 and 2017. A total of 2,978 DFA positive, negative and indeterminate post-mortem brain stem and/or cerebellum samples from rabies suspect animals or passive surveillance were tested in 14 participating laboratories (Table 2, S2A Fig). Samples originated from a wide variety of mammalian hosts (S2B Fig, S2 Table). As the LN34 assay was designed for pan-lyssavirus detection, samples were tested from across the world, including Africa, Asia, the Americas, Europe, and the Middle East, in the hopes of testing samples containing diverse viruses from the *Lyssavirus* genus and other pathogens causing similar clinical presentation to rabies. Indeed, although further testing was not required for rabies negative samples during this pilot study, two DFA negative samples were identified as canine distemper virus positive in the participating laboratory; no cross reactivity was observed in the LN34 assay for these samples.

#### Cut-off determination

Results of the pilot study were used to evaluate the accuracy of the LN34 assay and determine a threshold value for positive samples by ROC (Receiver Operating Characteristic) analysis. LN34 Ct values of DFA positive and negative samples were used for the analysis. Of the 1848 DFA negative samples, only 6 samples (0.3%) produced LN34 Ct values while the majority of the samples were not detected. All DFA positive samples produced LN34 Ct values. The area under the ROC curve (AUC) calculated for the LN34 assay was 1 (S3 Fig), providing evidence that the LN34 assay has excellent accuracy, as AUC > 0.9 indicates an excellent test [38].

ROC analysis can also be used to select a diagnostic cut-off for a given test [38,39]. Several common methods maximize both diagnostic sensitivity and specificity. However, diagnostic sensitivity and specificity are inversely related, and maximizing one will decrease the other. A threshold of Ct 35 was chosen to define positive samples (Ct ≤ 35), which corresponded to 99.81% (99.52–100) sensitivity and 99.84% (99.62–100) specificity (S3 Table). Based on the relationship between RNA copy number and Ct value described in the limit of detection analysis above (Fig 1A), LN34 Ct value of 35 corresponds to approximately 33 (95% confidence interval: 18 to 57) copies of positive control RNA. The threshold chosen for negative samples was lack of amplification. Any sample with LN34 Ct from 35–45 would, therefore, be inconclusive and must be retested (Table 4). This range for inconclusive results is further supported by the limit of detection and repeatability studies which revealed increased variability when LN34 Ct >35 (Figs 1B, 2A and S1A).

**Table 4:** LN34 assay diagnostic algorithm for post-mortem brain stem samples.

Result	LN34 Ct	β-actin Ct	Action	Interpretation
**Positive**	**≤ 35**	**Any**	**None**	**Lyssavirus RNA present**
**Negative**	**Not detected**	**≤ 33**	**None**	**No Lyssavirus RNA present**
Inconclusive	35–45	Any	Repeat or additional testing required	Contamination, inhibition, low virus load, or insufficient sample
Inconclusive	Not detected	> 33 or Not detected	Repeat or additional testing required	Insufficient sample, failed extraction or inhibition

Lack of LN34 amplification suggests lack of lyssavirus RNA but could also indicate failed RNA extraction, RNA degradation, PCR inhibition or assay failure. Therefore, a β-actin cut-off value was implemented to identify suitable samples. Rabies negative samples were defined as samples that did not exhibit LN34 Ct values (failure to amplify) and exhibited sufficient total RNA (as determined by the β-actin assay). The β-actin Ct value diagnostic cut-off was estimated based on all samples (DFA positive, negative, and indeterminate). A threshold of Ct 33 was chosen for suitable samples, as samples with Ct > 33 were very rare (0.5% of samples) and tended to show increased variability (Figs 2B and S1B). Thus, any sample that failed to amplify in the LN34 assay and had β-actin Ct ≤ 33 was defined as rabies negative. Any sample that failed to amplify in the LN34 assay and did not amplify in the β-actin assay or produced β-actin Ct > 33 was defined as inconclusive due to insufficient sample and must be re-tested (Table 4). LN34 Ct ≤ 35 was determined to be sufficient to define a rabies positive result, regardless of the β-actin outcome (Table 4). More extensive guidance on result interpretation and troubleshooting inconclusive samples can be found in S1 Text.

#### Preliminary pilot study results from the United States

The majority of pilot study samples originated from the United States and were tested in one of seven U.S. public health laboratories during the initial phase of assay evaluation (Table 5 and S2A Fig). A total of 2,120 U.S. samples were tested by the DFA test and LN34 assay. All samples were concordant with their original DFA test results using the diagnostic guidelines described for the LN34 assay (Table 4) except for one sample that was false positive by the LN34 assay (Table 5). This false positive sample was determined to have resulted from laboratory contamination. Sequencing of the LN34 amplicon from this sample revealed a rabies virus strain that is not known to circulate in the region where the animal was found but was used commonly as a positive control in the testing laboratory. Given these results, the LN34 assay had a diagnostic sensitivity of 100% (95% confidence interval (CI): 99.36–100%) and diagnostic specificity of 99.93% (95% CI: 99.62–100%).

**Table 5:** LN34 and DFA test results for 1,020 U.S. samples tested in U.S. laboratories.

	LN34 Positive	LN34 Negative	LN34 Inconclusive
**DFA Positive**	577	0	0
**DFA Negative**	1	1,474	0
**DFA Indeterminate**	23	45	0

#### Pilot study results from all laboratories

**Fig 3:**
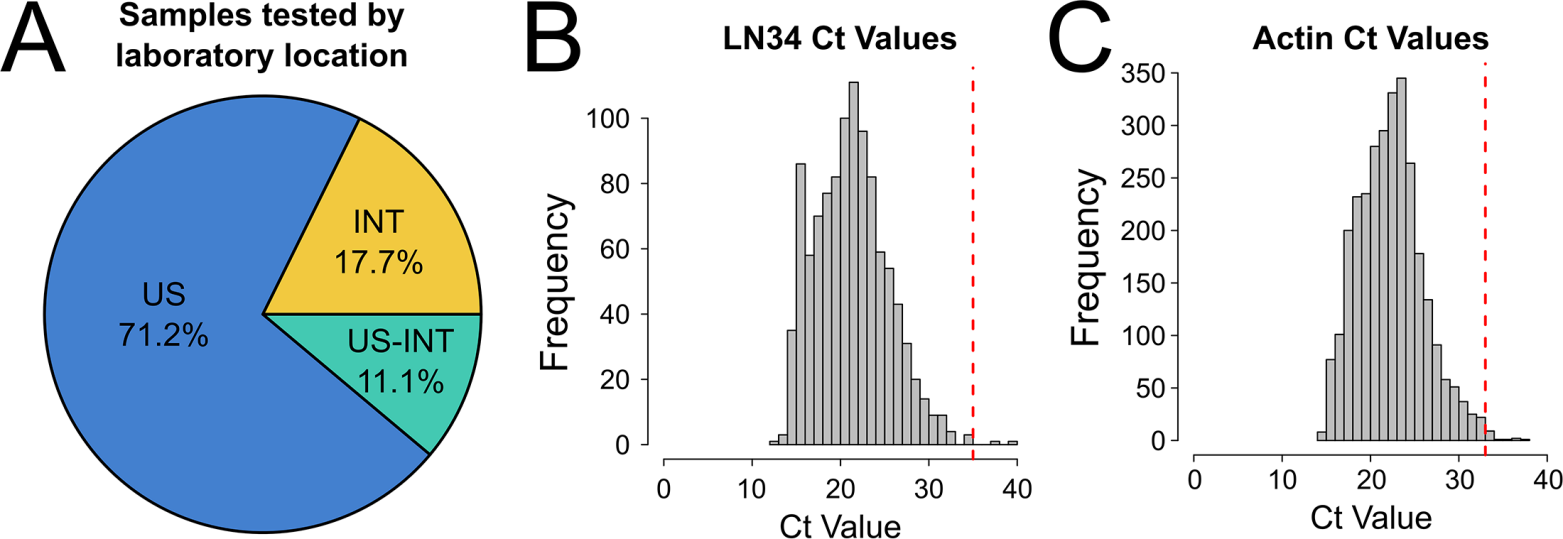
A multi-laboratory international evaluation of the LN34 assay. A. Proportion of samples originating and tested in the United States (US), originating and tested outside the U.S. (INT), or originating outside the U.S. and tested in the U.S. (US-INT). B. Frequency of LN34 Ct values for DFA positive samples. C. Frequency of β-actin Ct values for all 2,978 samples tested. Diagnostic cut-off values for positive samples (Ct 35 for LN34 and Ct 33 for β-actin) are highlighted by red vertical lines.

Upon successful implementation in United States laboratories, the LN34 assay was implemented in several international laboratories. In total, 28.8% of 2,978 samples tested by the LN34 assay and the DFA test originated from outside the United States, including a subset of international samples tested at CDC Atlanta (11.1% of total samples, Fig 3A).

A total of 1,049 (35%) of the samples tested in the pilot study were positive by the DFA test. For these samples, LN34 Ct values ranged from 12.35 to 37.40, with a mean of 21.22 (Fig 3B). No DFA positive sample was negative by the LN34 assay (Table 6 and S4 Fig). One DFA positive sample produced late amplification (LN34 Ct > 35) in the participating laboratory; it was tested in an international laboratory, had very low antigen distribution by the DFA test and produced Ct > 35 even upon repeated testing. The sample was unavailable for validation at CDC Atlanta. Together, these results reveal diagnostic sensitivity of the LN34 assay to be 99.90% (95% CI: 99.47–100%).

**Table 6:** LN34 and DFA test results for 2,978 samples tested in all laboratories during the LN34 assay evaluation.

	LN34 Positive	LN34 Negative	LN34 Inconclusive
**DFA Positive**	1,048	0	1
**DFA Negative**	3	1,842	3
**DFA Indeterminate**	29	51	1

In total, 2,978 samples were tested by the LN34 assay (Table 6). β-actin Ct values for all samples ranged from 14.00 to 37.41, with a mean of 22.28 (Fig 3C). Of the 1,848 DFA negative samples tested, three were positive by the LN34 assay (Table 6 and S4 Fig). One U.S. sample was consistent with contamination based on sequence analysis (as described above). The remaining two samples exhibited high LN34 Ct values very close to the Ct 35 cut-off (33.9 and 33.4). Both samples originated from international laboratories and were unavailable for repeat testing. Three DFA negative samples produced LN34 Ct > 35, defining them as indeterminate. All three samples were from international laboratories and were unable to be verified or further examined at CDC Atlanta. Based on these results, the diagnostic specificity of the LN34 assay was 99.68% (95% CI: 99.29–99.88%).

Participating laboratories were asked to test any samples that were indeterminate by the DFA test in their laboratory by the LN34 assay. In total, 81 of the 2,978 total samples were identified as indeterminate by the DFA test (Table 6). Of these, 29 DFA indeterminate samples were positive by LN34 assay and 51 were negative. Only one sample out of the 2,978 tested in the pilot study was indeterminate by both assays.

### Examination of LN34 assay performance in different sample types

**Fig 4:**
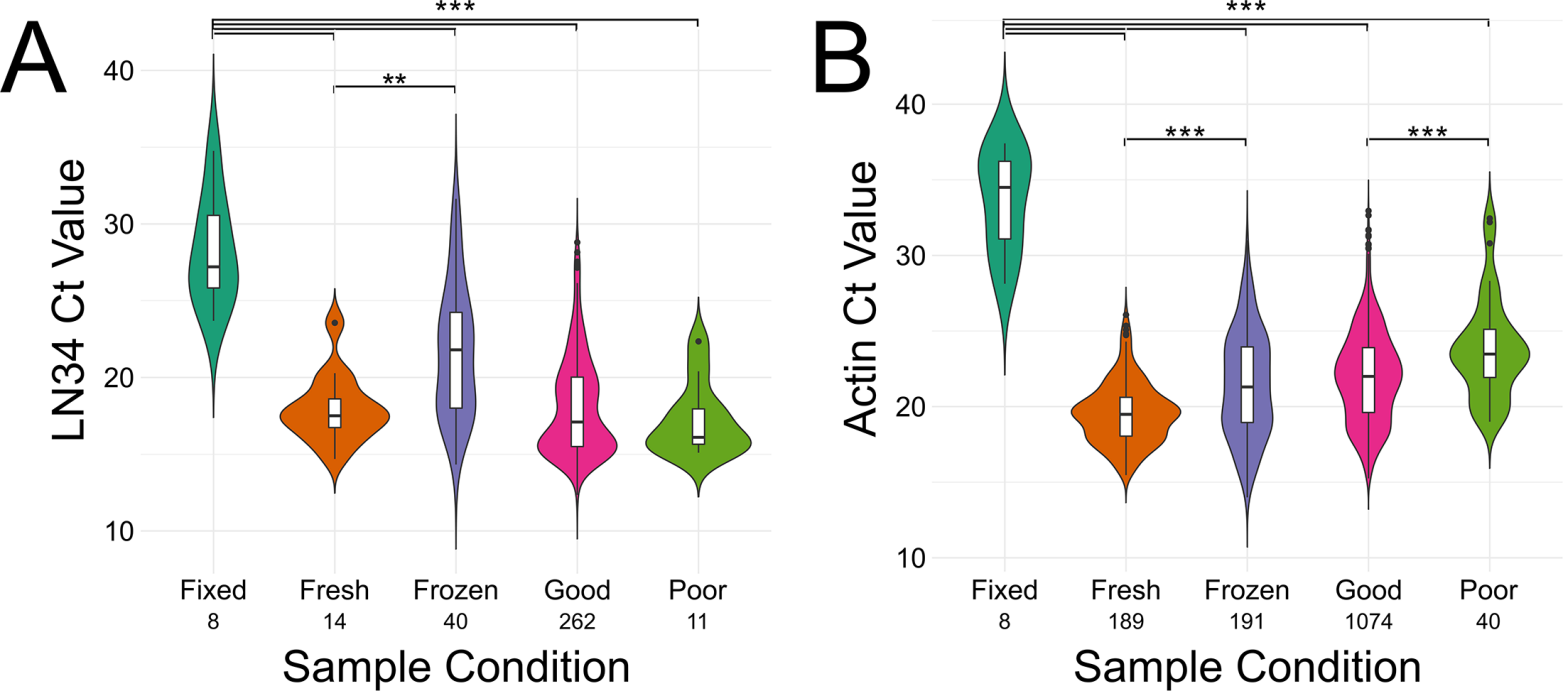
LN34 assay performance in different samples types. Violin plots showing LN34 (A) and β-actin (B) Ct values observed for brain samples of varying reported sample conditions. Overlaid boxplots show median (thick line), 25^th^ and 75^th^ quartiles. Whiskers indicate 1.5×(inter-quartile range). Data points beyond the whiskers are plotted individually. Data are only shown for samples that exhibited amplification. The number of samples for each condition is indicated under the sample condition. ** p <0.01, *** p < 0.001.

The goal of the international evaluation of the LN34 assay was twofold: to validate the LN34 assay for fresh and frozen brain samples that are appropriate for DFA testing and also to test the LN34 assay for samples unfit for DFA testing. Several laboratories reported samples unfit for DFA testing due to poor sample condition (e.g. liquefied or rotten) or inappropriate sample condition (e.g. fixed). Sample condition had a significant effect on LN34 (p < 0.0001, log-likelihood test) and β-actin Ct values (p < 0.0001, log-likelihood test). Formalin-fixed samples exhibited higher LN34 (p < 0.001, 9.5 ± 2.1 Ct increase) and β-actin (p < 0.0001, 11.9 ± 1.7 Ct increase) Ct values compared to other sample conditions (Fig 4). Additionally, frozen/archived samples produced slightly higher Ct values than fresh tissue (4.1 Ct increase in LN34 assay (p = 0.0005) and 1.9 Ct increase in the β-actin assay (p < 0.0001), Fig 4). Surprisingly, LN34 Ct values of samples described as poor condition were no different than those of samples described as good condition (p = 1.000, Fig 4A), demonstrating the potential of the LN34 assay for rabies diagnostics in poor quality tissue. Despite the significant effects of sample condition on Ct value, positive diagnoses were made for all positive samples using the current proposed diagnostic cut-off values. However, given the large increase in Ct values for fixed samples, a targeted evaluation with increased sample size must be performed to determine appropriate diagnostic cut-offs for these samples.

The LN34 real-time PCR assay was designed for use in all mammalian species. The host-species control β-actin real-time RT-PCR assay was adapted from Wakeley et al. (2005) [15] to detect a broad range of mammalian host species. Samples from over 60 host species were tested during the international evaluation, including many samples from major rabies reservoir species including dogs, raccoons, skunks, foxes, and many species of bats (S2 Table, S2B Fig). The exact number of species tested is not known, as the host was not always identified at the species level by participating laboratories. However, the β-actin assay was able to detect host mRNA in specimens from all species tested during the pilot study.

Together, these data suggest that the LN34 assay can be used for rabies diagnostics across mammalian species and several tissue conditions.

## Discussion

The international evaluation of the LN34 assay represents the largest evaluation of a rabies real-time RT-PCR assay to date, with 2,978 samples tested, 1,049 of which were DFA positive. Evaluation of the data revealed very low variability and excellent agreement with the gold standard test for post-mortem diagnosis of animal rabies, the DFA. The diagnostic sensitivity and specificity were 99.90% and 99.68%, respectively, which are similar to those of the DFA test and the direct rapid immunohistochemical test (dRIT) [2,10,40–44]. The diagnostic sensitivity and specificity values calculated here for the LN34 assay are conservative because they treat inconclusive results as false positives or false negatives. In practice, however, an inconclusive LN34 result simply identifies a sample that should undergo repeat testing. Furthermore, post-exposure prophylaxis is recommended in cases of an inconclusive rabies diagnostic result when case history, exposure and/or clinical symptoms suggest rabies [2], so the one DFA positive sample that was inconclusive by the LN34 assay would have resulted in treatment even if the DFA test had not been performed.

### The LN34 assay can be easily implemented in laboratories performing real-time PCR

Estimates suggest that, despite being preventable, rabies is responsible for approximately 60,000 human deaths worldwide each year, mostly in developing areas [45]. Many rabies endemic areas do not have the resources to implement DFA testing due to the requirements for continuous cold storage of samples, a fluorescent microscope, and highly trained technicians. The ability to store RNA samples at room temperature for PCR testing provides the possibility to increase rabies diagnostics in some areas. As many public health laboratories have adopted real-time PCR based diagnostics for identification of other pathogens, a reliable real-time PCR assay for rabies could be easily implemented at centralized reference laboratories with minimal equipment and training. Thirteen of the 14 participating laboratories in the pilot study were able to run the LN34 real-time RT-PCR assay using real-time PCR machines that were already in use for other diagnostics. Before sample testing, each new laboratory was required to validate LN34 assay performance and sensitivity against archived samples. During this testing phase, issues including PCR machine performance, contamination, and other quality issues were identified in several laboratories. Due to the simplicity of the assay and their previous experience with real-time PCR, all 13 laboratories were able to successfully address any issues quickly without hands-on training, highlighting the ease of implementation of the LN34 assay to diagnostic laboratories with real-time PCR experience.

The LN34 assay primer and probe sequences are published, making the assay standardized and freely available to laboratories across the world. The same cannot be said for DFA conjugates, which can vary in specificity and background between batches or manufacturers, and different conjugates and conjugate dilutions are used in different laboratories, making it hard to make a standardized DFA protocol. The cost of synthesis for the LN34 and β-actin primers and probes is minimal. The LN34 probe is most expensive to produce because it contains modified, locked nucleotide bases. Even still, synthesis using locked nucleotides is widely available, and large-scale production can reduce the cost considerably. However, new batches of real-time PCR reagents, positive control RNA, primers and probes should be validated by testing with samples with known Ct values to ensure assay performance. Furthermore, the LN34 assay has been validated for the reagents and real-time PCR machines mentioned here; laboratories may validate other reagents, methods, or machines as long as the sensitivity and diagnostic criteria are unaffected.

The use of positive and negative controls is an essential good practice for real-time PCR assays. The LN34 assay uses a standardized analyte positive control generated and distributed by CDC, which helped to monitor assay performance across different laboratories. Several laboratories also included positive and negative extraction controls prepared in their own laboratories in each assay run to help control the quality of results. The negative and positive control reactions, run in triplicate in each assay run, were good indicators of assay success and absence of buffer contamination. Another advantage of the LN34 assay is the detailed, objective records generated as real-time PCR machine outputs that can be examined at a later time or even remotely. Such records can help to quickly identify abnormal assay runs or inconsistencies in Ct value variability. These characteristics of the LN34 assay make it easy to set an international standard for rabies molecular testing as well as confirm and maintain such a standard across laboratories.

Based on the experience and findings of this multi-site evaluation of the LN34 assay, we have compiled a comprehensive set of publically available protocols and guidelines for any interested laboratory that includes trouble-shooting, advice, and interpretation of unusual results (https://www.protocols.io/private/e76bcdafd0578e49293e53c47fc84c2d; https://www.protocols.io/private/5c970341ebdf05cba17e58ebc16dff08; https://www.protocols.io/private/86d245bf034439795301b79dda52ee96, S1 Text).

### Detection of rabies virus in decomposed and fixed tissues

Previous studies have shown that PCR-based assays are capable of detecting rabies virus RNA in fixed tissues [46] and deteriorated tissues unfit for DFA testing [41,47–50]. We found that not only is the LN34 assay able to detect lyssavirus RNA in samples of poor condition but also average LN34 Ct values did not differ between samples of good condition and samples of poor condition. Furthermore, the result of the β-actin assay can be used to identify extensive RNA degradation, as an added control to avoid false negatives. These data suggest that the LN34 assay is appropriate for the identification of rabies virus in samples unfit for the DFA test due to sample condition. As these data were generated for different samples, future studies evaluating the effect of sample condition and storage on LN34 Ct value for the same sample will address the suitability of the LN34 assay for field-based rabies diagnostics and surveillance.

We also found that the LN34 assay is capable of detecting rabies virus RNA in rabies positive formalin-fixed tissues, which are untestable by the DFA test. The ability to test fixed tissues would eliminate the need for cold-chain and testing could be performed in parallel with immunohistochemistry. We observed that both LN34 and β-actin Ct values were significantly higher for formalin fixed samples than those of frozen brain stem samples. This finding is expected as RNA extraction from fixed tissue is less efficient, and may indicate that fixed tissue may require unique diagnostic cut-off values. As few formalin fixed samples were tested in the LN34 evaluation study, this question will have to be addressed by future work.

Real-time RT-PCR assays, including the LN34 assay [16], are capable of detecting rabies virus RNA in human antemortem tissues including skin and saliva [11,17,51], with increased sensitivity compared to virus isolation, at least in some cases [52]. Antemortem tissues have lower virus load and intermittent shedding, which would be expected to affect the LN34 Ct value. The diagnostic cut-off values for the LN34 assay presented here were based on data largely from frozen brain stem samples. Therefore, future directed studies with more formalin-fixed and antemortem samples should address whether these cut-off values are appropriate. Further investigation is also required to define the ability of the LN34 assay to rule-out rabies in these tissues types.

Lastly, although PCR-based assays such as the LN34 assay can produce results on suboptimal or deteriorated tissues, an important consideration in post-mortem rabies diagnostics is ensuring the tissue being tested is appropriate for rabies rule-out. The minimum recommended tissue for post-mortem rabies diagnosis by the LN34 assay is a full cross section of brain stem because virus may only show up in other tissues such as cortex, skin, saliva, hippocampus, and even cerebellum late in disease progression, intermittently or not at all [2–4,27]. As with the DFA test, we have confidence that a positive LN34 result in any tissue can be used to make a positive rabies diagnosis, but a negative result in a sample that is not representative of a full cross section of brain stem should be reported with caution as not sufficient to rule-out rabies.

### LN34 assay provided definitive results for DFA indeterminate samples

While most of the public health burden of rabies lies in areas with limited resources, rabies also has a sizable impact in areas with very few fatalities. Rabies diagnostics, prevention, and control is estimated to cost $245–$510 million each year in the United States [53]. Approximately 40,000 to 50,000 people in the United States undergo post-exposure prophylaxis per year, often costing over $3,000 per person [53]. Reducing the need for unnecessary post-exposure prophylaxis treatment could be achieved by decreasing the number of inconclusive and false positive diagnoses.

In this study, the LN34 assay was able to produce results for 80 out of 81 DFA indeterminate samples, including the identification of 29 presumptive positive samples missed by DFA. The false positive rate observed for the LN34 assay based on 1,848 DFA negative samples was 0.16% (0.32% including inconclusive results), providing strong evidence that these 29 LN34 positive samples were unlikely false positives. Furthermore, detection of rabies virus RNA by RT-PCR is an approved method to confirm the presence of rabies virus in samples indeterminate by the DFA test [2,4,27]. The high diagnostic specificity presented here suggests that the LN34 assay is reliable for the identification of rabies negative samples. Using both the LN34 assay and DFA test, only 1 sample out of 2,978 was inconclusive (0.03%), suggesting a potential for major improvement in rabies diagnostics in the future. The high specificity of the LN34 assay is reflected in the test’s ability to distinguish true negative samples from those that exhibit non-specific fluorescence. The high sensitivity of the LN34 assay may aid in the identification of weakly positive samples with very low antigen levels. In the future, the implementation of the LN34 assay could help rabies testing laboratories avoid false positive and false negative rabies diagnoses.

### LN34 assay improved rabies diagnostics in several laboratories

The LN34 assay was also able to identify issues with the DFA test in several laboratories. In each case, a discordant test result initiated repeat testing by both DFA and LN34 either at CDC Atlanta (for U.S. samples) or in the participating laboratory (for international laboratories). Almost all discordant samples were resolved upon repeat testing (S1 Table), and the combination of test results ensured accurate diagnosis in every case where sufficient sample was available for repeat testing.

One U.S. laboratory identified eleven DFA positive samples that were negative by the LN34 assay. A subset of these samples were sent to CDC Atlanta for confirmation, where both DFA and LN34 results were negative. This prompted a re-evaluation of the DFA method at the participating laboratory to avoid false positive diagnoses in the future. This case highlighted the ability of the LN34 assay to distinguish true negative samples from non-specific fluorescence observed in some cases by DFA. The LN34 assay provides a means to give labs the confidence to make a negative rabies diagnosis, even if some non-specific staining is observed in the DFA test.

In another U.S. laboratory, a false negative DFA result was identified when the LN34 assay produced a positive result for the same sample. Testing at CDC Atlanta produced positive results for both tests, but the antigen distribution was very low and sparse, especially in the cerebellum, which may have accounted for the false negative DFA result in the participating laboratory. This case highlights the high sensitivity and objective interpretation of LN34 assay results compared to the DFA test for samples with low virus load.

### LN34 assay discordance, errors, issues, and variability

A thorough investigation of all discordant results, failed assay runs, inconclusive results and reported issues revealed several important points about the LN34 assay and assay implementation. The majority of reported discordant samples were in agreement with DFA test results when repeated either at the testing laboratory or at CDC (S1 Table). Operator error, extraction failure and contamination were identified as the major causes of discordant samples, failed assay runs, or inconclusive results. Some laboratories reported higher instances of discordant results or issues than other laboratories (S1 Table), but for all laboratories, the number of issues and discordant results decreased, as expected, as operators become more experienced and accustomed to the assay. This finding suggests that each laboratory should test a comprehensive representative panel of known rabies positive and negative samples prior to implementation of the LN34 assay for rabies diagnostics. Each laboratory should ensure similar sensitivity, specificity, and variability as those reported here, to ensure diagnostic accuracy. We also suggest annual competency and proficiency testing and periodic regional trainings, as is currently suggested for the DFA test.

Several samples failed to produce amplification in the β-actin assay or produced β-actin Ct values above the cut-off of 33, indicating inefficient extraction, PCR inhibition, RNA degradation, or insufficient sample. Many of these samples produced positive β-actin results upon repeat testing, indicating loading error or extraction failure. For such samples, repeating RNA extraction is suggested, using more tissue. If PCR inhibition is suspected, testing serial dilutions of RNA to dilute out potential inhibitors is suggested. One laboratory reported abnormally high β-actin Ct values that were consistently above the cut-off of 33. A thorough investigation by the participating laboratory revealed that “fast mode” parameters proposed for the ABI 7500 FastDX real-time PCR machine produced reduced sensitivity in the β-actin assay, but not the LN34 assay, in their laboratory. While this issue has not been reported by other laboratories, we suggest using the “standard” mode and have adjusted the protocol to reflect this (https://www.protocols.io/private/86d245bf034439795301b79dda52ee96).

The high sensitivity of PCR-based molecular assays makes them inherently susceptible to contamination and false positive results. However, use of a one-step design helps to eliminate contamination as reverse transcription and PCR occur in a single step. In fact, very few cases of suspected or confirmed contamination were identified out of the 2,978 samples tested during the multi-site evaluation. However, the identification of contamination in one case by sequencing reinforces the idea that the utmost care must be taken during sample processing and reaction set up due. Guidelines on practices to avoid and recognize contamination are provided in the online protocol available at https://www.protocols.io/private/86d245bf034439795301b79dda52ee96 and in S1 Text.

The most common issue reported during the pilot study was appearance of Ct > 35 in one replicate well of a negative sample or no template control. This issue occurred in 1.07% ± 0.06 of negative samples tested in 3 reporting labs (based on 985 samples). When a subset of these samples were repeated, all samples produced negative results. The second most common issue reported was failure to amplify in one out of 3 replicates (occurrence: 0.15% ± 0.17 of positive samples tested based on 134 positive samples from 3 reporting labs). This error is most likely due to pipetting error or an issue with the real-time PCR machine.

Three laboratories participating in the international evaluation reported unusually high incidences of errors, Ct value variability, and/or failed or inappropriate amplification during their first assay runs. For instance, one laboratory reported LN34 amplification in 1/3 replicates (Ct > 35) in approximately 18% of 67 negative samples tested, while another laboratory observed amplification in 8 out of 8 negative samples in a single assay run. The results of such assay runs should be discarded, and the laboratory should not rely on the LN34 assay for rabies diagnostics until the sources of such issues are identified and addressed. These cases represent atypical assay performance and unusually large amounts of variability. Persistent issues or inconsistencies with the LN34 assay were uncommon and often indicated systemic issues in laboratory practices or the real-time PCR machine. In each case, consultation with CDC Atlanta and troubleshooting in the participating laboratory was sufficient to address the cause of the increased variability, inconsistent results or assay failure.

The diagnostic performance of the LN34 assay depends on maintaining low variability and high sensitivity wherever the assay is implemented. The positive control RNA acted as a reliable read-out of assay performance between laboratories and real-time PCR platforms. To achieve LN34 assay performance as presented here and identify potential sources of decreased sensitivity, laboratories implementing the LN34 assay should (1) test a panel of archived known positive and negative samples, (2) ensure that the LN34 assay can detect weakly positive or diluted positive samples, (3) ensure the LN34 assay can detect all lyssavirus variants circulating in the geographic area of interest, and (3) make certain performance and variability observed are comparable to those presented here and in other studies [16].

## Conclusions

The international evaluation presented here revealed the robustness, low variability, and excellent diagnostic specificity and sensitivity of the LN34 assay. Together, these data provide evidence that the LN34 assay is a reliable test for the identification of lyssavirus RNA in brain tissue. Even more, the LN34 assay played a role beyond confirming DFA test results in several cases. Discordant results between the LN34 assay and DFA test helped to identify systemic issues in a few laboratories. Two laboratories identified likely contamination during sample preparation and have re-evaluated their protocols to avoid cross-contamination in the future. The LN34 assay results identified a trend in false positive DFA results in one laboratory, which likely would have gone unnoticed if LN34 testing had not been performed. The LN34 assay also helped one laboratory avoid a false negative diagnosis in an atypical sample with low, sparse antigen distribution. Taken together, these observations suggest a role for the LN34 assay to improve routine animal rabies diagnostics as a primary post-mortem rabies diagnostic test, especially for samples unfit for DFA testing and in areas where DFA testing is not practical due to sample storage options.

## Supporting information

S1 Fig LN34 assay repeatability and reproducibility. A and B. Standard deviation (SD) between replicates of the same RNA sample from the same assay run plotted against average Ct value for that sample in the LN34 (A) and β-actin (B) assays. Vertical red lines indicate the diagnostic cut-off values for positive samples for each assay. Points are transparent; darker color indicates more overlapping points.
(TIF)

S2 Fig Multi-laboratory international evaluation of the LN34 assay. A. Proportion of samples originating from the most common host animals. Percent of samples where a host was identified is shown. B. Number of samples tested by each lab. Lab identities are removed except for CDC Atlanta.
(TIF)

S3 Fig Receiver Operating Characteristic (ROC) curve of LN34 assay results. Assay sensitivity is plotted against specificity for different Ct values (thick black line). The cut-off value of 35.07 is plotted on the ROC curve in blue; coordinates of this point are given in parenthesis. Area under the ROC curve (AUC, with 95% confidence intervals) is shown in the lower right corner. Gray line indicates a non-discriminant test.
(TIF)

S4 Fig Diagnostic results of the LN34 assay pilot study. Average LN34 Ct value is plotted against average β-actin Ct value for each sample. LN34 diagnostic results are shown by colored blocks based on diagnostic cut-off values of Ct 35 for LN34 and Ct 33 for β-actin. Points are colored based on their DFA results; positive samples are plotted in the graph on the left; negative samples are plotted on the right. Samples that failed to amplify are plotted at Ct 0. Points are transparent; darker color indicates more overlapping points.
(TIF)

S1 Text Result interpretation and troubleshooting guide. (DOCX)

S1 Table List of discordant results. (XLSX)

S2 Table List of host species reported during the LN34 evaluation study. (XLSX)

S3 Table Specificity and sensitivity associated with Ct value thresholds for the LN34 assay as determined by ROC analysis. Thresholds presented represent the local maximas for sensitivity and specificity based on the LN34 assay ROC curve (S3 Fig).
(XLSX)
