# Supplementary figures and images for: Multi-site evaluation of the LN34 pan-lyssavirus real-time RT-PCR assay for post-mortem rabies diagnostics
Source: PLoS One. 2018 May 16;13(5):e0197074. doi: 10.1371/journal.pone.0197074 (PMC5955534; doi:10.1371/journal.pone.0197074)

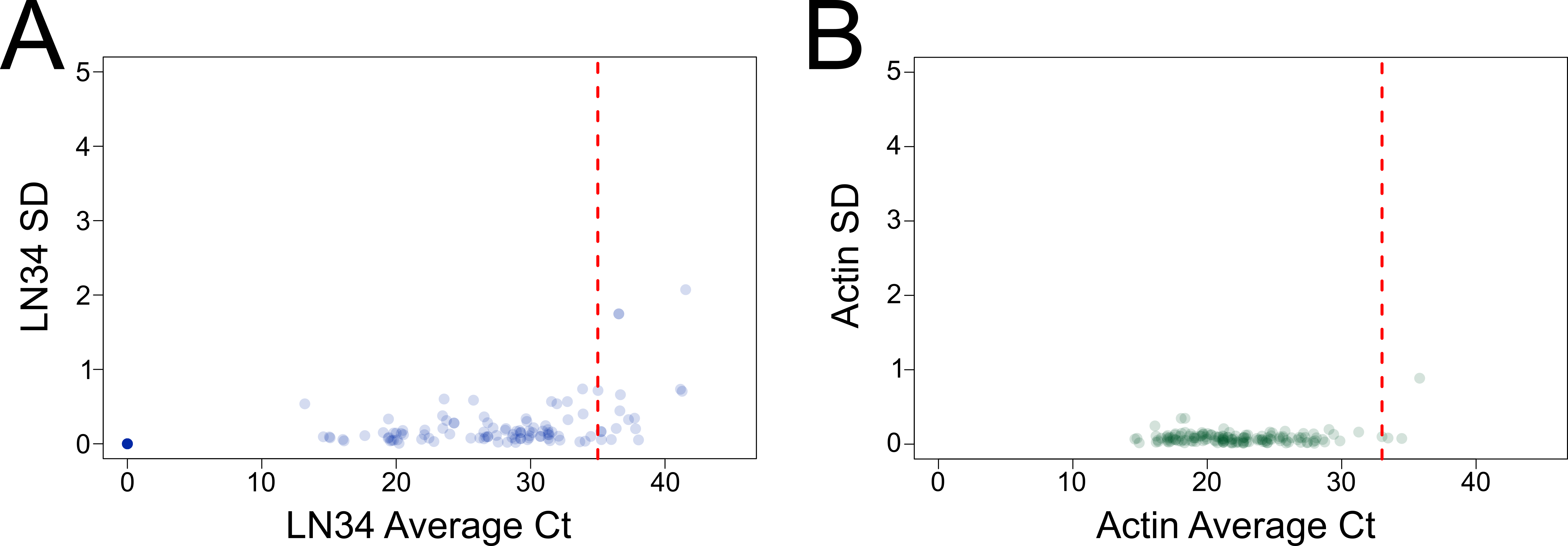

Supplement: S1 Fig — A and B. Standard deviation (SD) between replicates of the same RNA sample from the same assay run plotted against average Ct value for that sample in the LN34 (A) and β-actin (B) assays. Vertical red lines indicate the diagnostic cut-off values for positive samples for each assay. Points are transparent; darker color indicates more overlapping points. (TIF) [file pone.0197074.s001.tif]

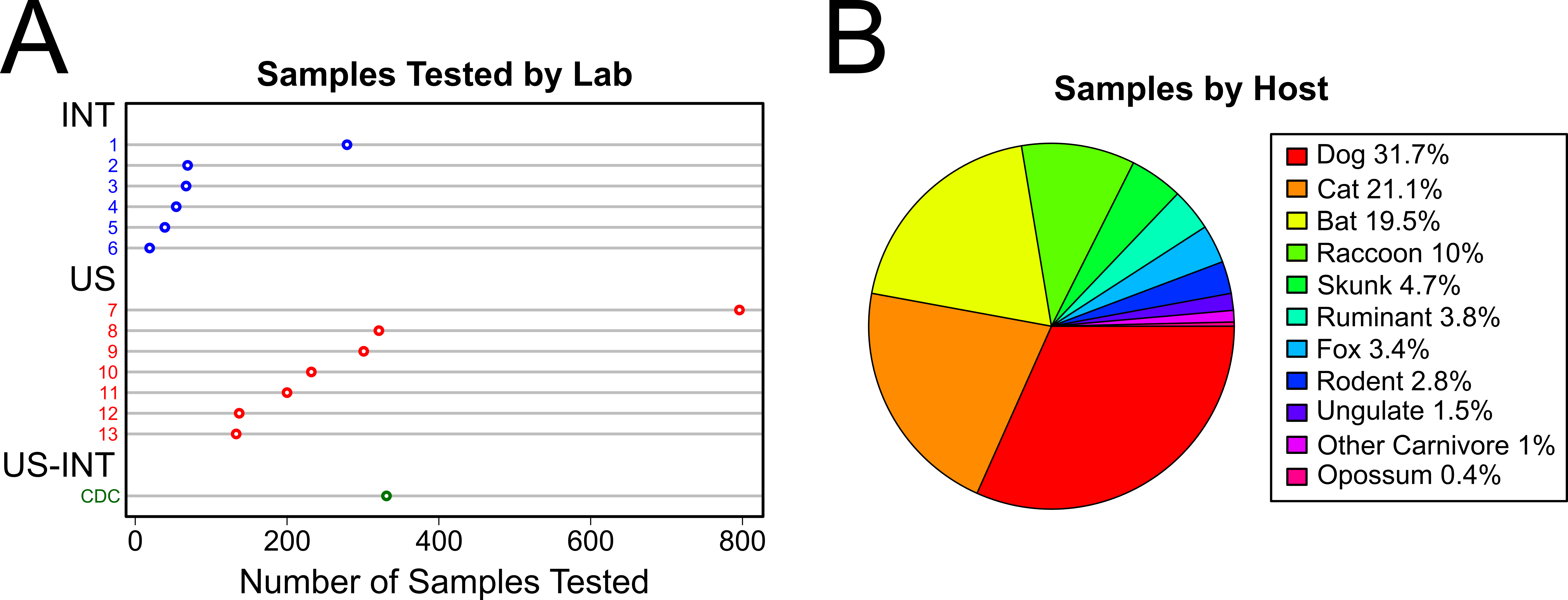

Supplement: S2 Fig — A. Proportion of samples originating from the most common host animals. Percent of samples where a host was identified is shown. B. Number of samples tested by each lab. Lab identities are removed except for CDC Atlanta. (TIF) [file pone.0197074.s002.tif]

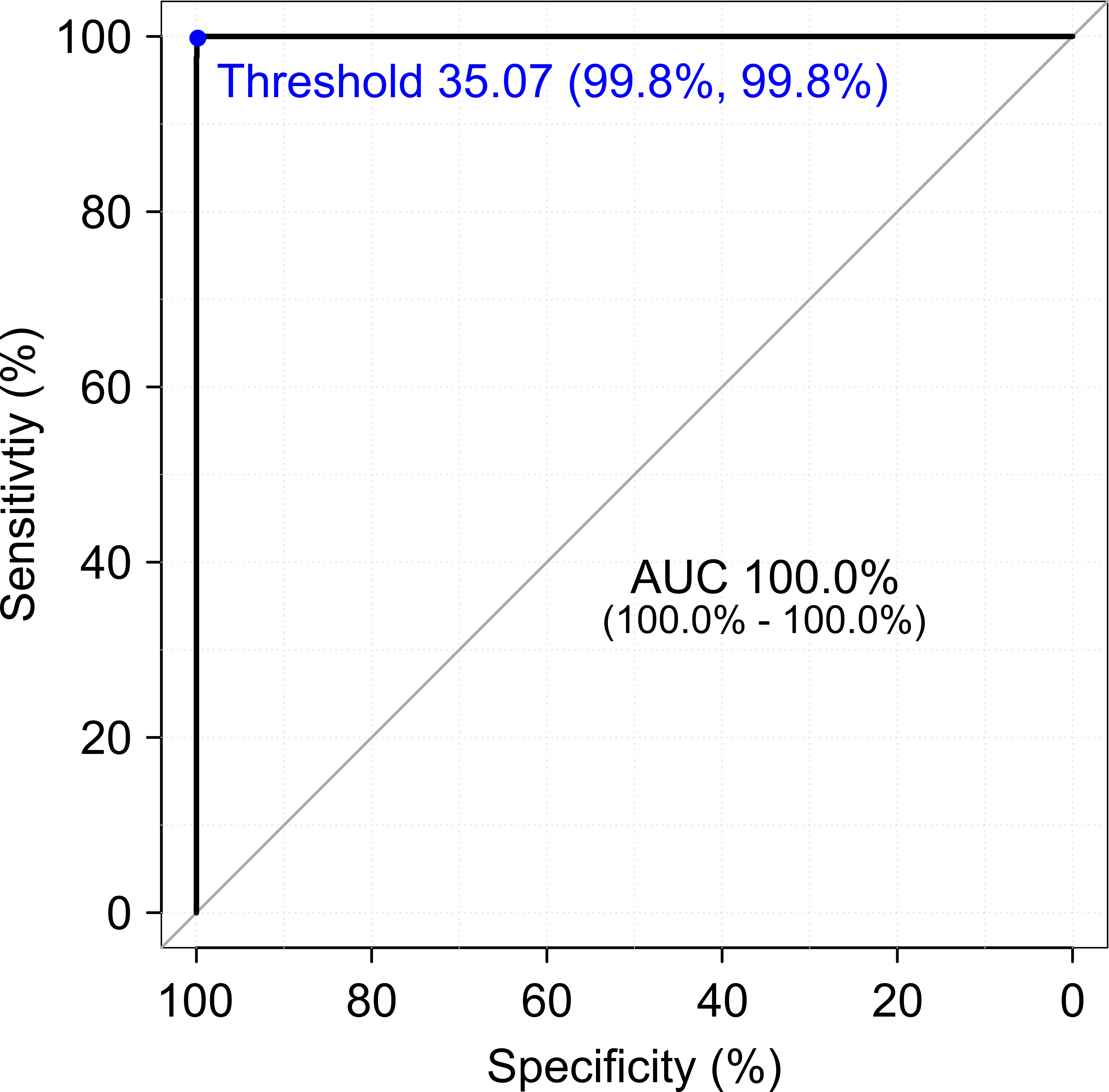

Supplement: S3 Fig — Assay sensitivity is plotted against specificity for different Ct values (thick black line). The cut-off value of 35.07 is plotted on the ROC curve in blue; coordinates of this point are given in parenthesis. Area under the ROC curve (AUC, with 95% confidence intervals) is shown in the lower right corner. Gray line indicates a non-discriminant test. (TIF) [file pone.0197074.s003.tif]

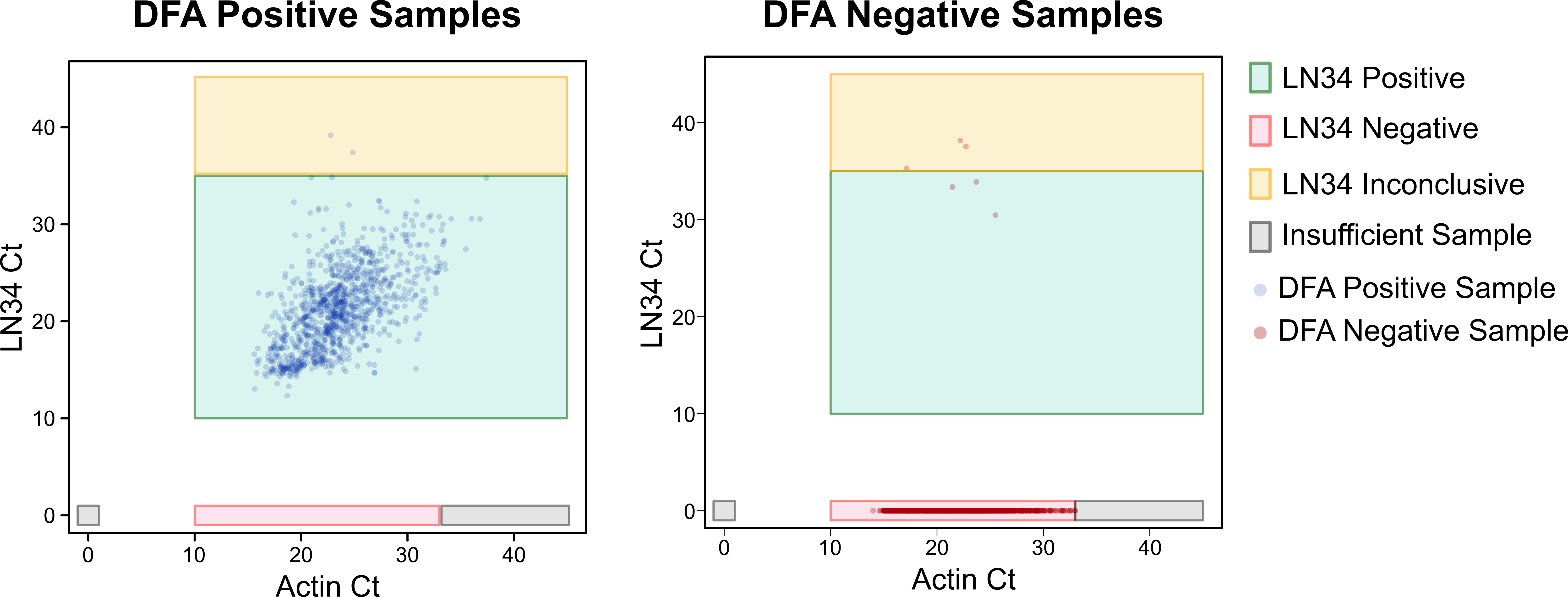

Supplement: S4 Fig — Average LN34 Ct value is plotted against average β-actin Ct value for each sample. LN34 diagnostic results are shown by colored blocks based on diagnostic cut-off values of Ct 35 for LN34 and Ct 33 for β-actin. Points are colored based on their DFA results; positive samples are plotted in the graph on the left; negative samples are plotted on the right. Samples that failed to amplify are plotted at Ct 0. Points are transparent; darker color indicates more overlapping points. (TIF) [file pone.0197074.s004.tif]
