# Supplementary material for: Multi-site evaluation of the LN34 pan-lyssavirus real-time RT-PCR assay for post-mortem rabies diagnostics
Source: PLoS One. 2018 May 16;13(5):e0197074. doi: 10.1371/journal.pone.0197074 (PMC5955534; doi:10.1371/journal.pone.0197074)
Supplement: S1 Text — (DOCX) [file pone.0197074.s005.docx]

**S1 Text. Result interpretation and troubleshooting guide**

**Example real-time PCR outputs:**

- - - 1. Successful assay run


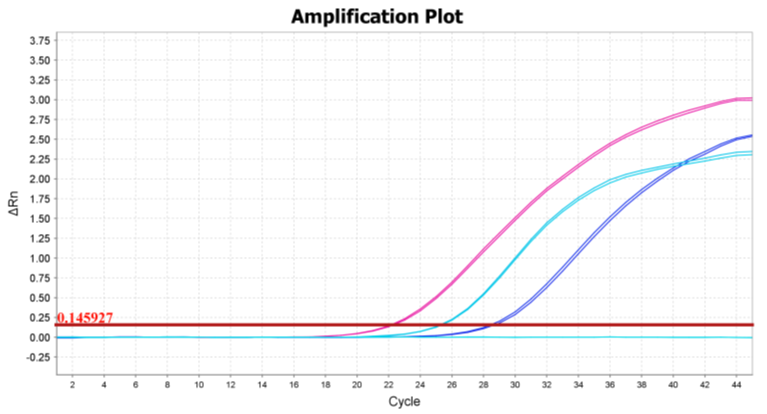


B.

A.


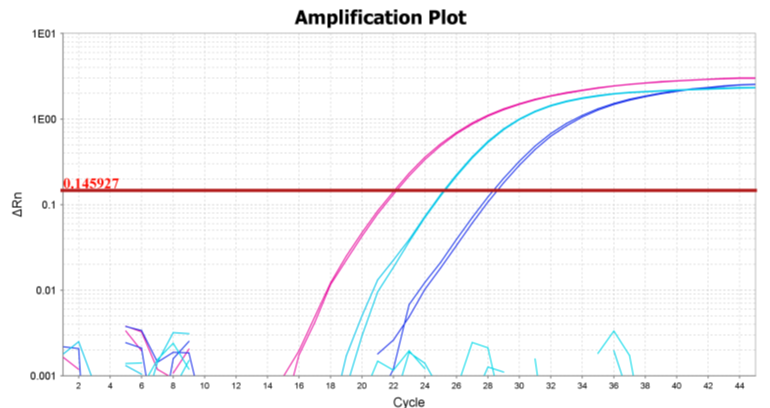


LN34

LN34

D.

C.


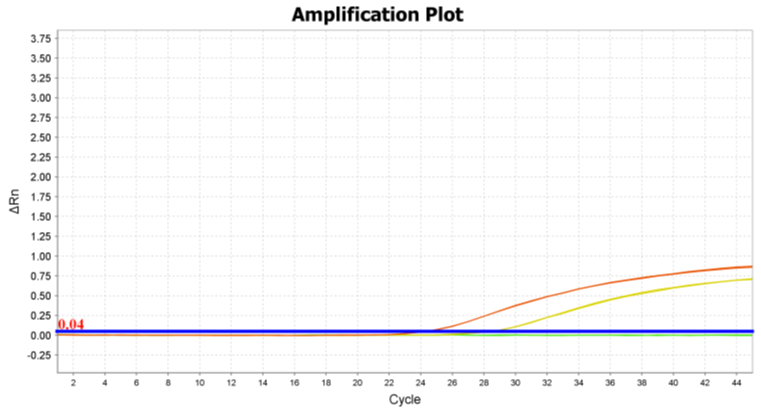

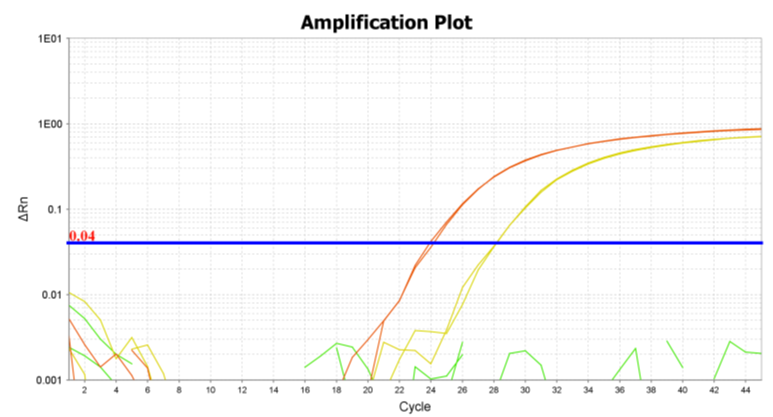


β-actin

β-actin

Representative images from a successful LN34 assay run on a ViiA7 real-time PCR machine. Results are shown for ΔRn plotted on a log scale (A and C) and linear scale (B and D). A and B show results from the LN34 assay for two samples (pink and dark blue) that have Ct values above and below the positive control RNA (cyan). Results for a third, negative sample is also shown in cyan, which can be seen as a flat line in B. The threshold was determined automatically and is shown by the red line at 0.146. The threshold for LN34 assay is typically around 0.15-0.25. C and D show results from the β-actin assay for two samples (orange and yellow) and the positive control RNA (green), which produced a negative result and no amplification in B. The threshold is shown in a blue line at 0.04. The threshold for the β-actin assay is typically observed around 0.04.

Key points:

- Successful amplification appears as an “S” shaped or sigmoidal curve when plotted on a linear scale (B and D).
- Negative samples should appear as a straight, flat line when plotted on a linear scale.
- β-actin curves typically level off lower than LN34 curves if plotted on the same graph.
- Viewing on a log scale allows for easy viewing of the Ct value, which is the point at which the curve crosses the threshold line.
- Viewing results in both views is suggested to identify possible anomalies or errors, see below for examples
  - - 1. Examples of abnormal amplification

A.

**
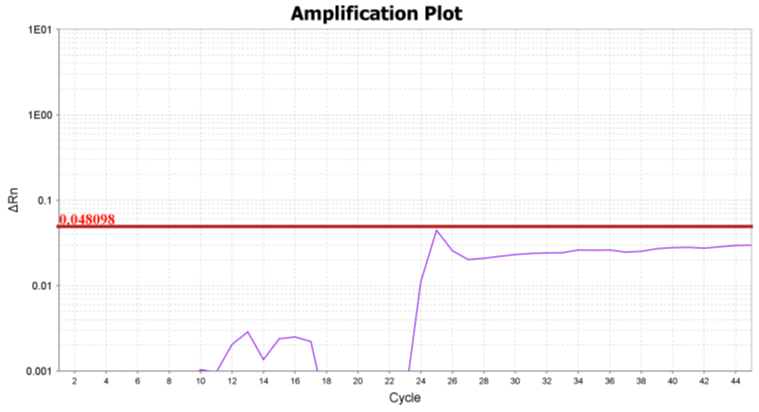
**
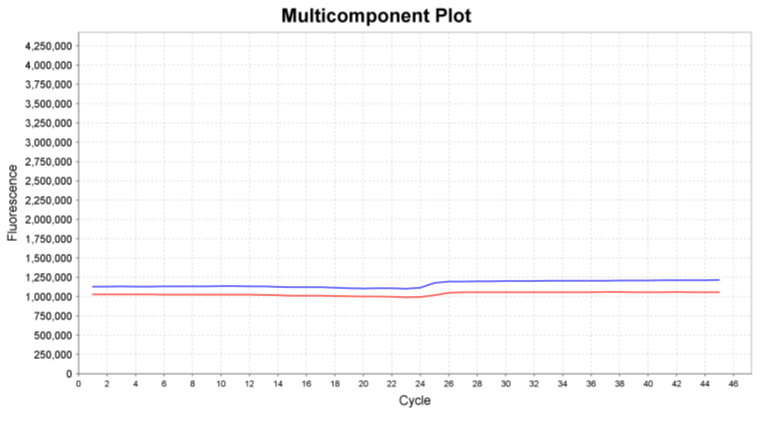


B.

**
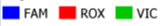
**

C.

**
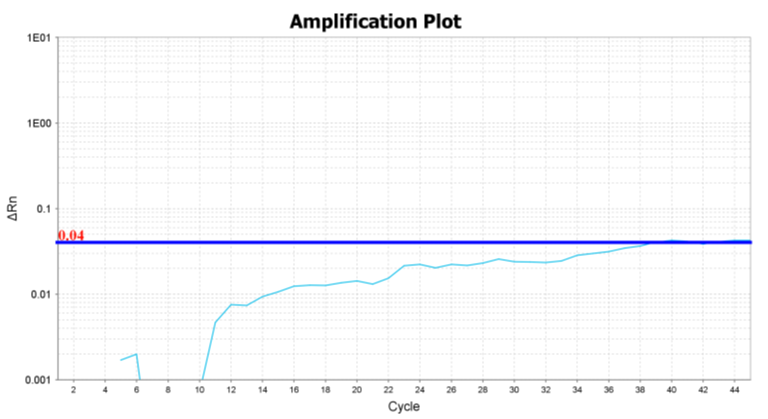

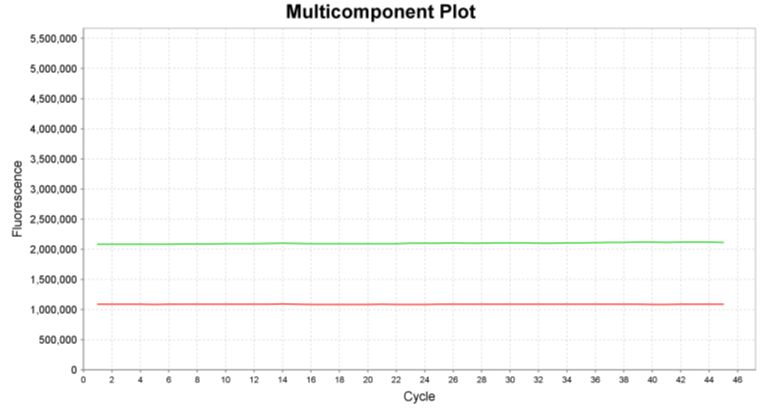
**

D.

**
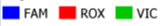
**

Representative images of several rare, atypical results from LN34 assay runs on a ViiA7 real-time PCR machine. Results are shown for ΔRn plotted on a log scale (A, C) and fluorescence vs. cycle (B, D). A and B are results from a single replicate showing very unusual signal in the ΔRn plot (A). The multicomponent plot (B) revealed that the sample was most likely negative (no increase in FAM fluorescence); however, there was an unusual blip in both the ROX and FAM fluorescence around cycle 25. Examination of all wells in the plate revealed the same blip. The run was deemed unsatisfactory and was repeated. C and D show results from a replicate in the β-actin assay that gave Ct 39; however, the signal in the ΔRn plot (C) was very unusual and the sample should not be called positive. The multicomponent plot (D) revealed that this sample was likely negative and should be repeated.

Key Points:

- Familiarity with the real-time PCR output graphs can help identify some issues
- Ensure the amplification curves look normal for all samples; do not simply copy Ct values
- Viewing the multicomponent plot can give insight missed in the ΔRn plot and vice versa.
  - - 1. Using the multicomponent plot to identify a contaminated well

B.

A.


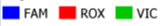

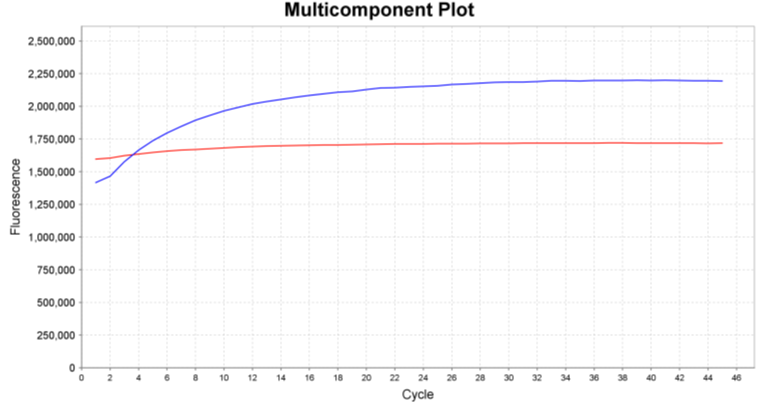

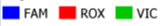

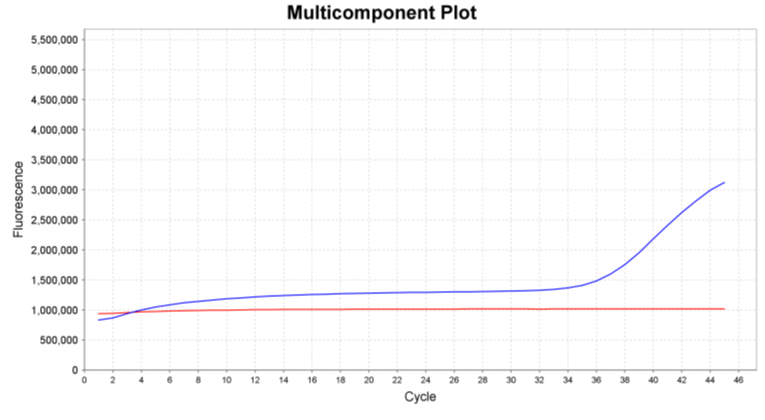

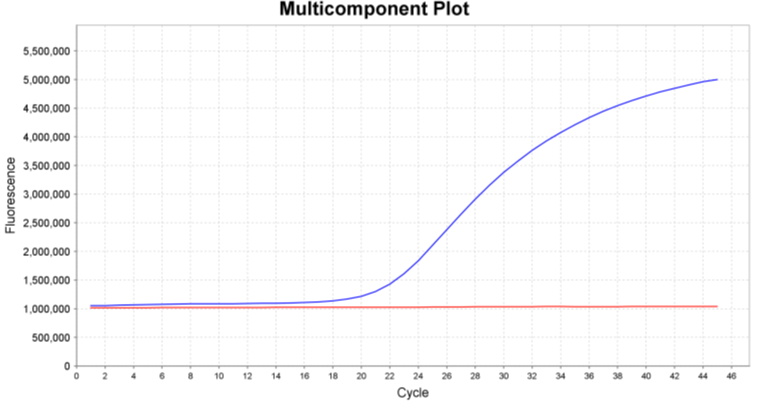

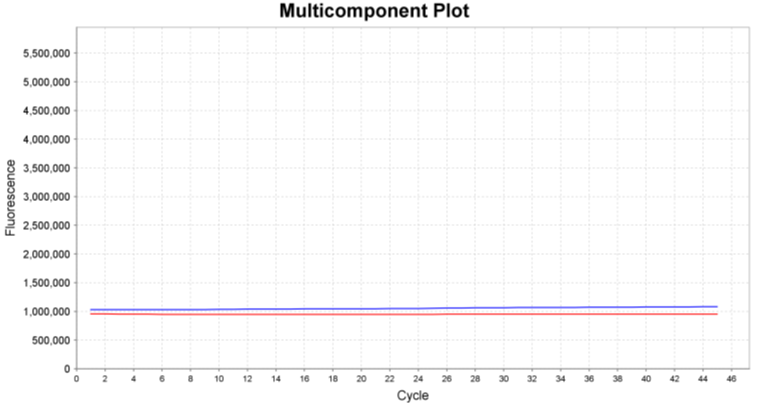

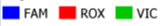

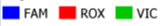

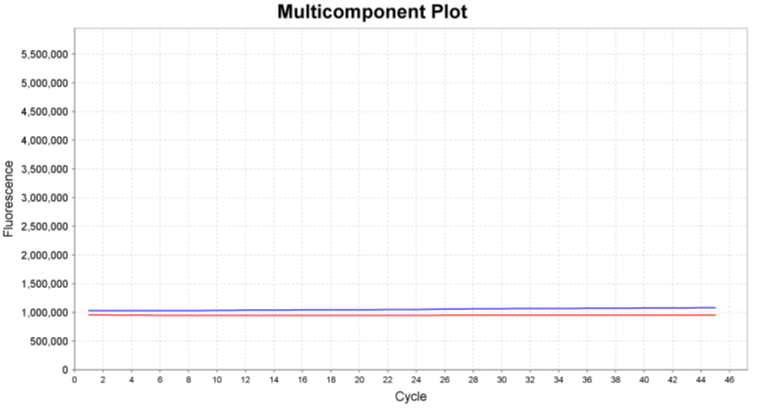


C.

D.

Representative images from a successful LN34 assay run on a ViiA7 real-time PCR machine.

Multicomponent plots show the fluorescence at each cycle for FAM (LN34), VIC (Actin) and ROX (control present in the buffer). ROX levels should stay flat across all cycles. A typical positive sample is shown in A; FAM fluorescence increases starting at cycle 18 for this sample. The amplification is observed as a sigmoidal curve. A typical negative sample is shown in B, where the FAM level stays flat across all cycles.

C and D show atypical results for a positive (C) and a negative (D) sample. The increase in FAM fluorescence during cycles 1 – 8 is atypica for normal rabies positive samples. The increase in FAM fluorescence after cycle 34 in C represents true amplification, based on the cycle and shape of the curve. The ROX fluorescence was also not completely flat, which can be easily observed in D. Both C and D were results from the same well (well C2 in this case) on different days. Replicates of the same samples run in wells C1 and C3 did not exhibit the increase in fluorescence during cycles 1 – 8. Taken together, this suggested contamination of well C2. Cleaning per the manufacturer’s suggestion was sufficient to resolve the problem.

**Identifying possible issues:**

1. Unacceptable variability

The LN34 assay should exhibit low variability between replicates, assay run, operator, and laboratory. Observation of high variability (>5 Ct difference) between replicates of the same sample suggests atypical assay performance and that the sample should be repeated. Observation of such a phenomenon across several samples (such as shown in the table below) or across assays runs may indicate systemic issues in laboratory practices or real-time PCR machine. In such a case, the entire assay run must be repeated. Consultation with CDC and troubleshooting in the participating laboratory is necessary to address the cause of the increased variability, inconsistent results or assay failure if such issues persist.

The table below shows a real result from a single assay run during implementation of the LN34 assay. Replicate Ct values showing unusual variability are highlighted. In this assay run, not a single negative sample, including the no template control (NTC) reaction was not detected (ND) in all replicates of the LN34 assay. In this assay run, the LN34 assay results are unacceptable. The amount of variability in the LN34 assay results suggests possible contamination during assay set-up, which may not be noticeable in the β-actin assay because all samples are positive. In such a case, the entire run must be repeated using the same RNA.

1. Contamination

The high sensitivity of PCR-based assays make them inherently susceptible to contamination. The best way to avoid contamination is through strict adherence to good laboratory practices, including frequently changing gloves, keeping tubes closed, manipulating samples one at a time, cleaning work surfaces between samples, and keeping samples from contacting PCR reagents. One way to accomplish this last point is to prepare PCR mastermixes in a location separate from samples. Fresh gloves should be worn to avoid contaminating PCR reagents with sample or positive control RNA. After adding mastermixes to the plate, the plate can be moved to a second location where RNA can be added.

Knowing how to identify potential contamination is also important. Below is example data from a real LN34 assay run showing reagent contamination. In this case, the assay run was deemed invalid because all three of the no template control (NTC) wells showed amplification. This meant the assay run must be repeated. However, careful examination of the results can reveal more information. The Ct values of all Sample 1, Sample 2, and NTC replicates were similar to each other, suggesting contamination of one of the PCR reagents. Reagents are typically contaminated with a small amount of RNA, producing high Ct values like those observed in this case. This issue was resolved when the assay was re-run using a new aliquot of PCR reagents and the same RNA, resulting in no amplification in any replicates of Sample 1, 2, or the NTC. Making aliquots of reagents is suggested to minimize the risk of contamination and to avoid having to throw away large volumes of costly reagents in the case that they are contaminated.

Cross contamination of samples can be more difficult to identify. If a laboratory suspects that sample contamination has occurred, repeat testing should be performed. It is suggested that a new brainstem sample is collected from the original tissue or a new sample is requested from the submitter. If the laboratory would like to confirm contamination, sequencing of the viral RNA can be performed. This can be helpful if the contaminant strain is very different from the naturally circulating strains (such as a control virus used in the laboratory). Sequencing can also be used to determine if two isolates are identical, as when contamination is suspected between two samples processed at the same time, but this approach can become costly and may be uninformative if the sequences are very similar.

If sample contamination with the positive control RNA is ever suspected, run the LN34 assay amplicons on an agarose gel to determine if the band runs at 165 bp (lyssavirus RNA) or 127 bp (positive control RNA).
